# Low offspring survival in mountain pine beetle infesting the resistant Great Basin bristlecone pine supports the preference-performance hypothesis

**DOI:** 10.1371/journal.pone.0196732

**Published:** 2018-05-01

**Authors:** Erika L. Eidson, Karen E. Mock, Barbara J. Bentz

**Affiliations:** 1 Wildland Resources Department, Utah State University, Logan, Utah, United States of America; 2 Ecology Center, Utah State University, Logan, Utah, United States of America; 3 USDA Forest Service Rocky Mountain Research Station, Logan, Utah, United States of America; Umeå Plant Science Centre, Umeå University, SWEDEN

## Abstract

The preference-performance hypothesis states that ovipositing phytophagous insects will select host plants that are well-suited for their offspring and avoid host plants that do not support offspring performance (survival, development and fitness). The mountain pine beetle (*Dendroctonus ponderosae*), a native insect herbivore in western North America, can successfully attack and reproduce in most species of *Pinus* throughout its native range. However, mountain pine beetles avoid attacking Great Basin bristlecone pine (*Pinus longaeva*), despite recent climate-driven increases in mountain pine beetle populations at the high elevations where Great Basin bristlecone pine grows. Low preference for a potential host plant species may not persist if the plant supports favorable insect offspring performance, and Great Basin bristlecone pine suitability for mountain pine beetle offspring performance is unclear. We infested cut bolts of Great Basin bristlecone pine and two susceptible host tree species, limber (*P*. *flexilis*) and lodgepole (*P*. *contorta*) pines with adult mountain pine beetles and compared offspring performance. To investigate the potential for variation in offspring performance among mountain pine beetles from different areas, we tested beetles from geographically-separated populations within and outside the current range of Great Basin bristlecone pine. Although mountain pine beetles constructed galleries and laid viable eggs in all three tree species, extremely few offspring emerged from Great Basin bristlecone pine, regardless of the beetle population. Our observed low offspring performance in Great Basin bristlecone pine corresponds with previously documented low mountain pine beetle attack preference. A low preference-low performance relationship suggests that Great Basin bristlecone pine resistance to mountain pine beetle is likely to be retained through climate-driven high-elevation mountain pine beetle outbreaks.

## Introduction

Many insect herbivores are undergoing geographic range shifts as a result of climate change [1,2], often leading to new, more frequent, or restored associations with alternative potential host plant species. The preference-performance hypothesis [3] predicts that female insects use cues (i.e., visual, olfactory, or gustatory stimuli) to assess plant suitability for their offspring and preferentially oviposit on host plants that will support optimal offspring performance (survival, development and fitness). Strong correspondence between oviposition preferences and offspring performance is evident in many cases [4]. In plant and insect populations with either limited or disrupted prior contact, however, the lack of time for oviposition preferences to adapt can result in mismatched preference-performance relationships [5]. For example, females may initially avoid a potential host plant species because they fail to recognize that the plant is suitable for their offspring [6–8]. However, if at least some females choose the plant species and their offspring perform well, increased preference may be anticipated [7,9]. Alternatively, if offspring performance on the plant species is poor, natural selection should act to maintain low preference if other hosts are available. Preference-performance relationships are therefore useful for predicting future plant vulnerability to phytophagous insects.

The mountain pine beetle (MPB, *Dendroctonus ponderosae* Hopkins, Coleoptera: Curculionidae, Scolytinae) is a native insect herbivore that is responding to climate change with shifting geographic range margins. Over the past several decades, rising temperatures have been linked to MPB range expansion northward into the boreal forest [10] and increased MPB population success at high elevations [11,12]. MPBs attack and kill most species of pine (*Pinus*) throughout western North America by boring through the bark to feed and reproduce in the phloem. Lodgepole pine (*Pinus contorta* Douglas) is considered to be a primary host tree species due to its high abundance in the current MPB range. However, climate-driven MPB population growth at high elevations intersects landscapes where pine species such as whitebark pine (*P*. *albicaulis* Engelm.), limber pine (*P*. *flexilis* James), and bristlecone pines (three closely-related species in *P*. subsection *Balfourianae*) occur in greater abundance than or in lieu of lodgepole pine. Climate change is expected to continue to support MPB success at high elevations throughout this century [12,13]. Given sustained climate-driven MPB pressure, the future vulnerability of high-elevation pine species depends largely on MPB attack preference and offspring performance in these hosts.

Records show that high-elevation MPB outbreaks have occurred intermittently throughout the past century during particularly warm and dry periods, killing both high-elevation whitebark and limber pines [14,15]. Whitebark and limber pines also experienced high levels of MPB-caused mortality in recent climate change-driven outbreaks [16,17], indicating favorable MPB attack preference for both high-elevation pine species. MPB offspring performance in both tree species has also been found to be favorable, with studies showing that MPB offspring perform equally well or better in whitebark and limber pines as compared to lodgepole pine [18–21]. Successful MPB attacks and reproduction in whitebark and limber pines raise concerns that other high-elevation pine species may also be vulnerable to extensive MPB-induced mortality with warming temperatures.

Great Basin (GB) bristlecone pine (*P*. *longaeva* Bailey), a member of the *Balfourianae* pine group, is a high-elevation pine species found in Utah, Nevada, and California. Several recent studies have shown that unlike whitebark and limber pines, it is generally not attacked by MPB. In surveys of mixed stands of GB bristlecone and limber pines, MPBs avoided GB bristlecone pine even when >34% of co-occurring limber pines were attacked and killed [22]. In two-way choice tests, the volatile organic compounds of GB bristlecone pine foliage repelled female MPBs (the host-selecting sex) even when the alternative offered choice was just clean air [23]. Finally, in no-choice tests where female MPBs were confined to exposed areas of tree boles, extremely few beetles attacked GB bristlecone pine, even when placed on freshly cut tree sections with no capacity to induce defensive reactions [24].

GB bristlecone pine is well-known for being the longest-lived non-clonal organism in the world, capable of living for thousands of years despite adverse growing conditions [25]. Currently, GB bristlecone pine occupies high-elevation sky islands between ~2,800 and 3,500 meters, but its elevational range likely fluctuated over time, expanding down slope during cool ice ages and retreating upslope during interglacial warming [25]. These elevational transitions between glacial periods may have provided windows of time where the range of areas that were climatically favorable for bark beetles overlapped GB bristlecone pine forests. Evidence suggests that GB bristlecone pine most recently existed at low elevations between 25,000 and 11,000 years ago, forming extensive low-elevation forests with limber pine before retreating upslope in the early Holocene epoch [25,26]. Limber pine remains a common associate of GB bristlecone pine today, but unlike GB bristlecone pine, limber pine is readily attacked by MPB and also supports offspring development. MPB offspring performance in GB bristlecone pine has not been tested, and could have important consequences for the future vulnerability of this keystone tree species.

The purpose of this study was to investigate MPB reproduction and offspring performance in GB bristlecone pine. We evaluated MPB mating success, fecundity, brood production, and offspring fitness in freshly cut GB bristlecone pine relative to limber and lodgepole pines, two host species known to be well-suited for MPB propagation [21,27]. Additionally, we tested for evidence of variation in reproductive success among beetles from different regions by using MPBs from two geographically-separated populations, located within and outside the current range of GB bristlecone pine. This research clarifies the preference-performance relationship between MPB and GB bristlecone pine and aids in predicting future GB bristlecone pine vulnerability to MPB.

## Materials and methods

### Study system

Preference-performance relationships in MPBs are complex because adult beetles must successfully invade a host tree before mating, oviposition, and offspring development can occur in the phloem. Female MPBs use visual, olfactory, and gustatory cues to locate, assess, and select host trees for colonization, after which they proceed with gallery construction beneath the bark [28]. During this process, attacking MPBs emit aggregation pheromones to recruit other conspecifics to join the attack [28]. Pheromone-mediated, synchronous, high-density attacks are required to exhaust attack-activated induced defenses in living host trees (i.e., toxic resin production), but these defenses are considered to be absent in cut trees [29]. In addition to attack-induced defenses, host trees generally also invest in constitutive defenses that are pre-formed and always present in the tree. Constitutive defenses can include obstructive bark compounds, resin duct characteristics, and permanently expressed phloem toxins [30]. Unlike induced tree defenses, many constitutive defenses are presumably retained in felled trees. Because MPB offspring are sessile and restricted to one host for their entire development, the ability of females to choose susceptible hosts that will support offspring development, and to avoid hosts that are detrimental for their offspring, is vitally important for MPB population success.

The reason for MPB avoidance of GB bristlecone pine is unclear. Although induced defenses have not been studied in GB bristlecone pine, recent research indicates that GB bristlecone pine phloem contains constitutive toxins that are over eight times more concentrated than those in co-occurring high-elevation limber pine [22]. These compounds have been associated with tree defense in other pines [31], but the direct effects of constitutive GB bristlecone pine traits on MPB adults and their offspring have not been tested.

### Sourcing and handling of host tree materials and parent MPBs

**Fig 1:**
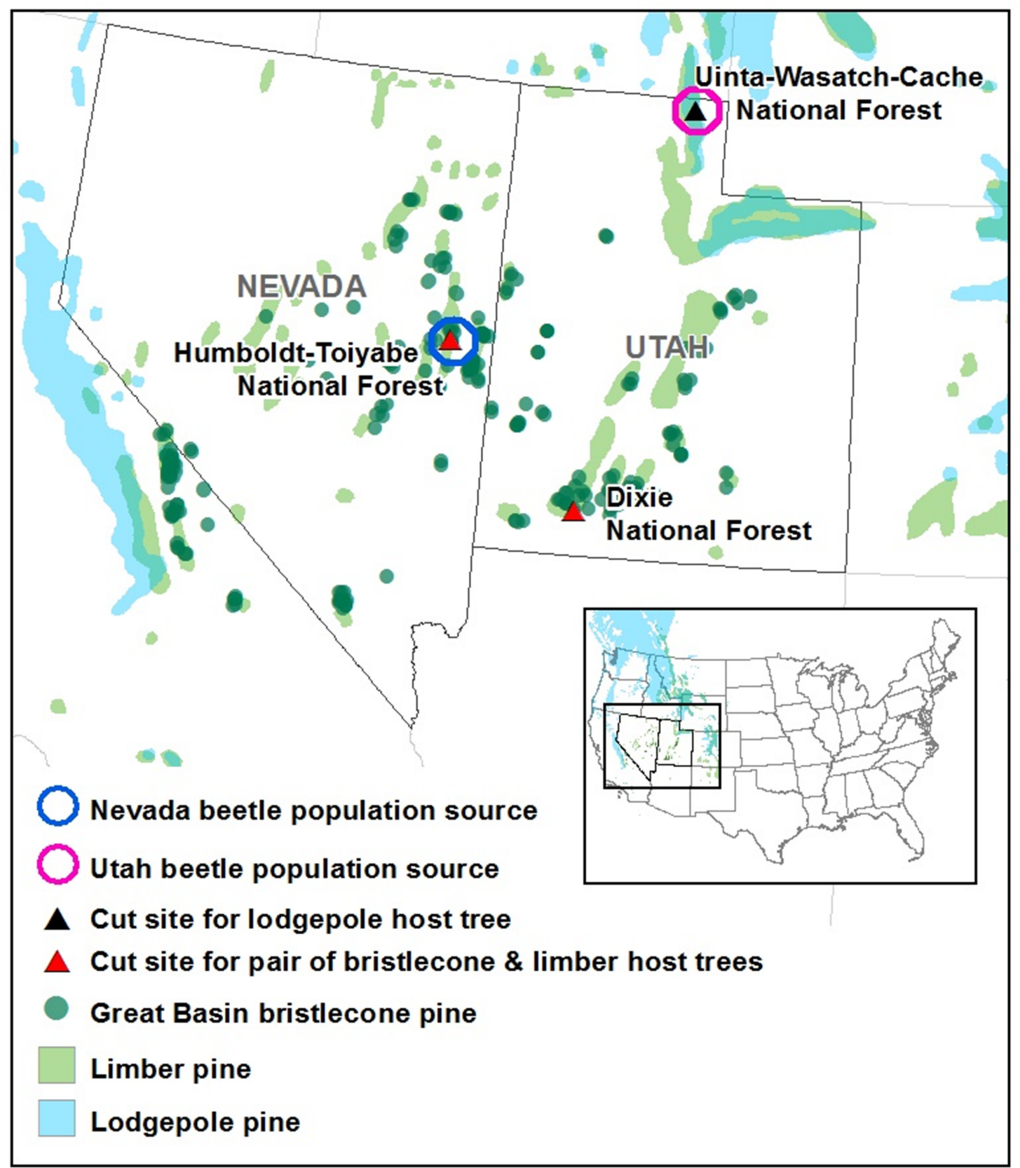
Locations of parent mountain pine beetle population sources and tree harvest sites. Two mountain pine beetle-infested limber pines were cut from Nevada and one infested lodgepole pine was cut from Utah to obtain two populations of unmated mountain pine beetle adults for use in tests. Live, uninfested Great Basin bristlecone and limber pines were cut from the Humboldt-Toiyabe National Forest and the Dixie National Forest sites (a single tree of each species from each site), and one live, uninfested lodgepole pine was cut from the Uinta-Wasatch-Cache National Forest site. Shown on the map are observed points of Great Basin bristlecone pine occurrence (data from Bentz *et al*., 2016 [22]) and limber and lodgepole pine distributions [32].

We obtained uninfested, cut bolts of GB bristlecone, limber, and lodgepole pines to be manually infested with MPBs by harvesting two live GB bristlecone-limber pine pairs in August, 2015 and one healthy lodgepole pine in September, 2015 (cut five trees total, all trees ~30–35 cm dbh). Paired trees were growing in close proximity (< .5 km apart) under similar growing conditions and were similar in vigor (live crown ratio and crown density). One bristlecone and limber pine pair (BR-HT and LM-HT, respectively) was cut from the Humboldt-Toiyabe National Forest, NV (39°10'52.9"N, 114°37'11.7"W), and the second pair (BR-DX and LM-DX) was cut from the Dixie National Forest, UT (37°28'46.1"N, 112°43'46.6"W). The lodgepole pine (LP-UWC) was cut from the Uinta-Wasatch-Cache National Forest, UT (41°52’30.2"N, 111°29’29.7"W) (Fig 1). We cut ~40 cm long bolts from the lower boles of each tree and sealed bolt ends with paraffin wax to reduce desiccation. We recorded the average phloem thickness (mm) calculated from two measurements taken at opposite sides of each bolt. Bolts were stored just above 0° C for between 26 and 81 days before use.

To test for evidence of variation in MPB performance among beetles from different locations, we collected unmated adult MPBs from two population sources in different geographic regions and natal host tree species. The first group of MPBs was collected by felling two infested limber pines from a mixed stand of GB bristlecone and limber pine on the Humboldt-Toiyabe National Forest, NV (39°09'41.9"N, 114°36'54.2"W) (Fig 1) in August, 2015. The second group of MPBs was obtained from one newly infested lodgepole pine cut from a lodgepole-subalpine fir (*Abies lasiocarpa* Hook) stand on the Uinta-Wasatch-Cache National Forest, UT (41°52’30.2"N, 111°29’29.7"W) (Fig 1) in September, 2015. Unlike the NV site, the UT site was located outside of GB bristlecone pine range.

MPBs infesting the NV limber pines had several potential advantages for reproductive success in GB bristlecone pine over those infesting the UT lodgepole pine. First, relative to MPBs raised in lodgepole pine, beetles that develop in limber pine have been found to be larger, more fecund, and are hypothesized to have superior detoxification systems [21,27], which may improve their ability to contend with the high concentrations of phloem compounds in GB bristlecone pine. Additionally, although GB bristlecone pine is not currently attacked by MPB, it may have been successfully colonized in the past. Because the NV beetle population was collected from within current GB bristlecone pine range, whereas the UT beetle population was collected from outside its range (Fig 1), the NV beetle population may have had increased opportunity in the past for host adaptation. Our rationale for choosing these two MPB populations was to investigate whether the potentially superior fitness and possible increased opportunity for adaptation would allow the NV beetles to achieve greater offspring performance in GB bristlecone pine cut bolts as compared to the UT beetles.

Cut bolts (~30–70 cm in length) of the infested trees from NV and UT were labeled by site and transported to the U.S. Forest Service Rocky Mountain Research Station in Logan, UT. Bolt ends were sealed with paraffin wax to reduce desiccation, and bolts were placed in enclosed rearing containers for brood to complete development. Newly emerged adults were collected twice daily and stored in petri dishes with moistened filter paper at approximately 4°C until use, up to ~2 weeks. Male and female beetles were identified and separated using secondary sex characters on the seventh tergite [33]. The pronotal width (mm), hereafter referred to as the ‘size’ of each intended parent beetle, was measured using an ocular microscope scale to facilitate subsequent investigations of the relationship between parent size and offspring performance in contrasting host tree species.

### Manual infestation of tree bolts

We manually infested eight cut bolts from each of the five host trees (bristlecone pines BR-HT and BR-DX, limber pines LM-HT and LM-DX, and lodgepole pine LP-UWC) with MPB adults according to protocols established by Bentz et al. (2001) [34]. Four bolts of each tree were infested with NV beetles and the remaining four were infested with UT beetles. Using a power drill (bit size ~5 mm diameter), we initiated parent galleries to a depth of approximately 2 cm along the bottom cross sectional plane of each bolt (Panel (a) in S1 Fig). We avoided placing galleries directly over wounds or branch stubs, but otherwise maintained an equal gallery spacing of ~6 cm to standardize intra-specific competition among offspring. Between 13 and 19 galleries were initiated for each bolt. One female followed by one male MPB were manually inserted into each initiated gallery, after which the hole was screened to prevent the pair from exiting (Panel (b) in S1 Fig). After a bolt was completely infested (Panel (c) in S1 Fig), it was turned to its original upright orientation within the tree so that the screened entrance holes were at the base and the inserted beetles were facing upwards to construct vertical parent galleries as in natural attacks. Infested bolts were placed in incubators (Percival Scientific, Inc., Perry, Iowa) at 22.5°C to facilitate brood development. Each incubator included a mixture of bolts from different host trees.

### Mating success and fecundity

After egg hatch, developing MPB larvae feed horizontally outward from the vertical parent gallery, often in increasingly meandering patterns that intersect other larval galleries. Over time, this feeding pattern, in addition to fungal growth, can obscure individual larval galleries and eggs in the phloem. As development progresses, fecundity (i.e., the total number of eggs laid per parent pair) becomes difficult to measure. For this reason, we refrigerated one NV and one UT beetle-infested bolt of each of the five host trees on day 26 after manual infestation in order to halt further reproductive development and assess the mating success and fecundity of each pair [35]. We peeled the bark and phloem from the 10 refrigerated bolts to expose galleries and eggs in the phloem. For each manually infested parent gallery in refrigerated bolts, we recorded (1) mating success or failure, (2) total parent gallery length (cm), and (3) the total sum of hatched eggs (larval galleries) plus eggs that had not yet hatched. Mating was recorded as ‘unsuccessful’ for parent pairs that had either died, emerged through the bark without constructing a vertical gallery, or constructed a vertical gallery with no evidence of oviposition. Mating was recorded as ‘successful’ for parent galleries with evidence of larval galleries or eggs. Unhatched eggs were often found at the end of the parent gallery and were assumed to be oviposited later than the hatched eggs at the beginning of the parent gallery.

### Offspring emergence

The remaining three UT and three NV beetle-infested bolts of each host tree were individually caged with screen (S2 Fig) and remained at 22.5°C. Adult offspring began emerging from the infested bolts approximately 50 days after manual infestation. We collected newly emerged offspring from each bolt once daily for days 50–100 after infestation and continued to do so until either five consecutive days had passed with no new offspring emergence or day 150 was reached. At that time, bolts were moved to refrigeration to halt any additional development and fungal growth.

We recorded the size and sex of each emerged offspring. After bolts were moved to refrigeration, we peeled the bark and phloem and recorded individual parent gallery lengths (cm). Parent gallery scoring of xylem was easily visible, but offspring development left minimal xylem scoring and individual larval galleries were difficult to distinguish in the degraded phloem. In bolts that were peeled 26 days after infestation in the mating and fecundity study described above, >93% of unsuccessful parent galleries were <10 cm in length. Therefore, parent galleries that were <10 cm were recorded as ‘unsuccessful’ and parent galleries >10cm were recorded as ‘successful’.

### Statistical analyses

Data were analyzed with generalized linear mixed models using GLIMMIX (SAS Studio version 9.4). All models used Laplace maximum likelihood estimation, and least squared means estimates for multiple comparisons were calculated using Tukey’s range test on the inverse link scale [36]. Because one GB bristlecone pine and one limber pine each were cut from two sites, but the lodgepole pine was the only host tree cut from the third site, we had an unbalanced factorial design for considering host tree species and host tree site. For this reason, rather than grouping host trees by species, we considered all five host trees individually in analyses to create a balanced, one-way factorial design with five levels. This approach also allowed us to assess intraspecific differences between the two GB bristlecone pines and the two limber pines. Specific model information is summarized in Table 1 and described in detail below. Distributional assumptions were tested for each variable using residual plots and tests of normality (S1 Supporting Information).

**Table 1:** Summary of model information for all statistical tests.

Test	Variable	Experimental unit	Fixed effects	Random Effects	Response Distribution	Link Function
**1**	male parent size	beetle	MPB pop.	-	Gaussian	identity
**2**	female parent size	beetle	MPB pop.	-	Gaussian	identity
**3**	male parent size for NV beetles	beetle	host tree	-	Gaussian	identity
**4**	female parent size for NV beetles	beetle	host tree	-	Gaussian	identity
**5**	male parent size for UT beetles	beetle	host tree	-	Gaussian	identity
**6**	female parent size for UT beetles	beetle	host tree	-	Gaussian	identity
**7**	phloem thickness	bolt	host tree	-	gamma	log
**8**	MF probability of parent mating success	parent gallery (all)	host tree; MPB pop.	-	binary	logit
**9**	MF parent gallery length	parent gallery (successful)	host tree; MPB pop.	-	Gaussian	identity
**10**	MF total fecundity	parent gallery (successful)	host tree; MPB pop.	-	negative binomial	log
**11**	OE parent gallery length	parent gallery (successful)	host tree; MPB pop.	bolt nested within host tree and MPB pop.	Gaussian	identity
**12**	OE avg. number of offspring / successful parent gallery	bolt	host tree; MPB pop.	bolt nested within host tree and MPB pop.	Poisson	log
**13**	OE male offspring size	beetle	host tree; MPB pop.	bolt nested within host tree and MPB pop.	Gaussian	identity
**14**	OE female offspring size	beetle	host tree; MPB pop.	bolt nested within host tree and MPB pop.	Gaussian	identity

Abbreviations and definitions: MPB pop.–mountain pine beetle population source; NV–Nevada; UT–Utah; MF–mating and fecundity study; OE–offspring emergence study; parent gallery (all)–all parent galleries tested; parent gallery (successful)–only tested parent galleries where beetles were considered to have successfully mated; avg.–average. MPB pop. levels = NV or UT; host tree levels = bristlecone pines BR-HT and BR-DX, limber pines LM-HT and LM-DX, and lodgepole pine LP-UWC.

We tested for size differences between parent beetles from NV and parent beetles from UT (Table 1, tests 1–2). To verify that similarly-sized parents within each population had been used to infest all host trees, we also tested for parent beetle size differences across host trees for each beetle population (Table 1, tests 3–6). Due to female-biased sexual size dimorphism in MPBs [37], male and female parent beetle sizes were tested separately. MPB brood production has been shown to be positively related to phloem thickness [28,38], therefore we tested for differences in the average phloem thickness of each bolt (Table 1, test 7). Because of missing data due to human error, measurements from only four of the eight total lodgepole pine bolts were used in phloem thickness tests.

Analyses of mating and fecundity data were conducted using individual parent galleries because there was only one bolt replicate of each host tree-beetle population source combination. We tested for differences in the probability of parent mating success (Table 1, test 8), individual parent gallery length (Table 1, test 9), and total fecundity (sum of larval galleries and unhatched eggs) per parent gallery (Table 1, test 10). All initiated parent galleries were used in test 8, but only successful parent galleries were used in tests 9 and 10. We initially investigated the effects of parent beetle sizes on the response variables but found weak and/or inconsistent relationships. Therefore, parent beetle sizes were ultimately excluded from final model analyses.

Results from offspring emergence experiments were analyzed using an added random-effects factor to account for offspring grouping within bolts (Table 1, tests 11–14). Offspring emergence continued for longer in some bolts than others, and relatively few offspring emerged after day 100. To standardize offspring emergence collection periods across all bolts, only offspring that emerged between day 50 and day 100 after infestation were included in model calculations. We tested for differences in individual parent gallery length (Table 1, test 11), the total number of emerged offspring / the number of successful parent galleries for each bolt, hereafter ‘average total number of emerged offspring per successful parent gallery’ (Table 1, test 12), and offspring size; tested separately for each sex (Table 1, tests 13–14). For tests 11 and 12, only parent galleries marked as ‘successful’ were used. Again, parent beetle sizes did not strongly influence the response variables, so parent beetle sizes were ultimately excluded from final model calculations for these tests.

## Results

### Parent beetle sizes and bolt phloem thickness

**Fig 2:**
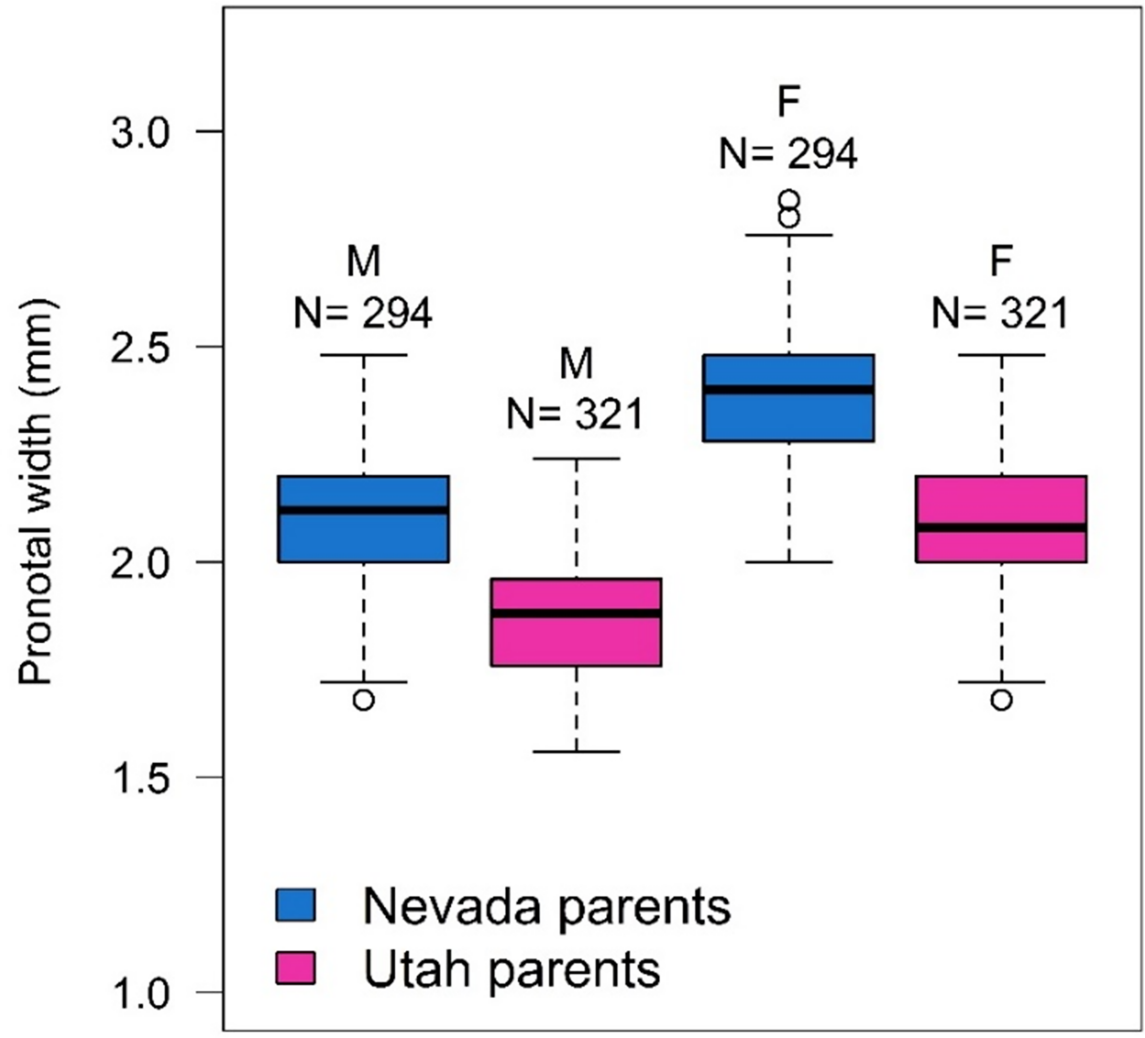
Male (M) and female (F) parent mountain pine beetle size from the Nevada and Utah populations. Shown are the median (solid middle line), 25th and 75^th^ percentiles (top and bottom of box), ± 1.5 x the interquartile range (whiskers), and all outliers (points). N = number of beetles in each category.

Parent MPBs from the NV population were significantly larger than parent beetles from the UT population for both males (*F*_1,613_ = 512.52, *P* < .0001) and females (*F*_1,613_ = 727.66, *P* < .0001) (Fig 2). Within each beetle population, statistical tests indicated that there were several unintended size differences between parent beetles randomly chosen to infest each host tree. In the NV population, male parent size differed across infested host trees (*F*_4,289_ = 3.27, *P* = 0.0121), but female parents were similarly-sized in all host trees (*F*_4,289_ = 0.56, *P* = 0.6913). NV male parents infested into bristlecone pine BR-HT were smaller than those infested into bristlecone BR-DX and limber pine LM-DX (adjusted *P* values for multiple comparisons = 0.0108 for BR-HT and BR-DX and 0.0402 for BR-HT and LM-DX). In the UT population, both male and female parent size differed across infested host trees (*F*_4,316_ = 8.04, *P* < .0001 for UT males; *F*_4,316_ = 7.80, *P* < .0001 for UT females). UT male parents infested into lodgepole pine LP-UWC were larger than those in all other host trees (adjusted *P* values range 0.0042 to < .0001 for multiple comparisons). UT female parents were also the largest in lodgepole LP-UWC, and were significantly larger than those infested into all other host trees except limber LM-HT (adjusted *P* values range 0.0058 to < .0001 for multiple comparisons). Additionally, UT female parents infested into limber LM-HT were larger than those infested into LM-DX and BR-HT (adjusted *P* values for multiple comparisons = 0.0049 for LM-HT and LM-DX and 0.0458 for LM-HT and BR-HT).

**Fig 3:**
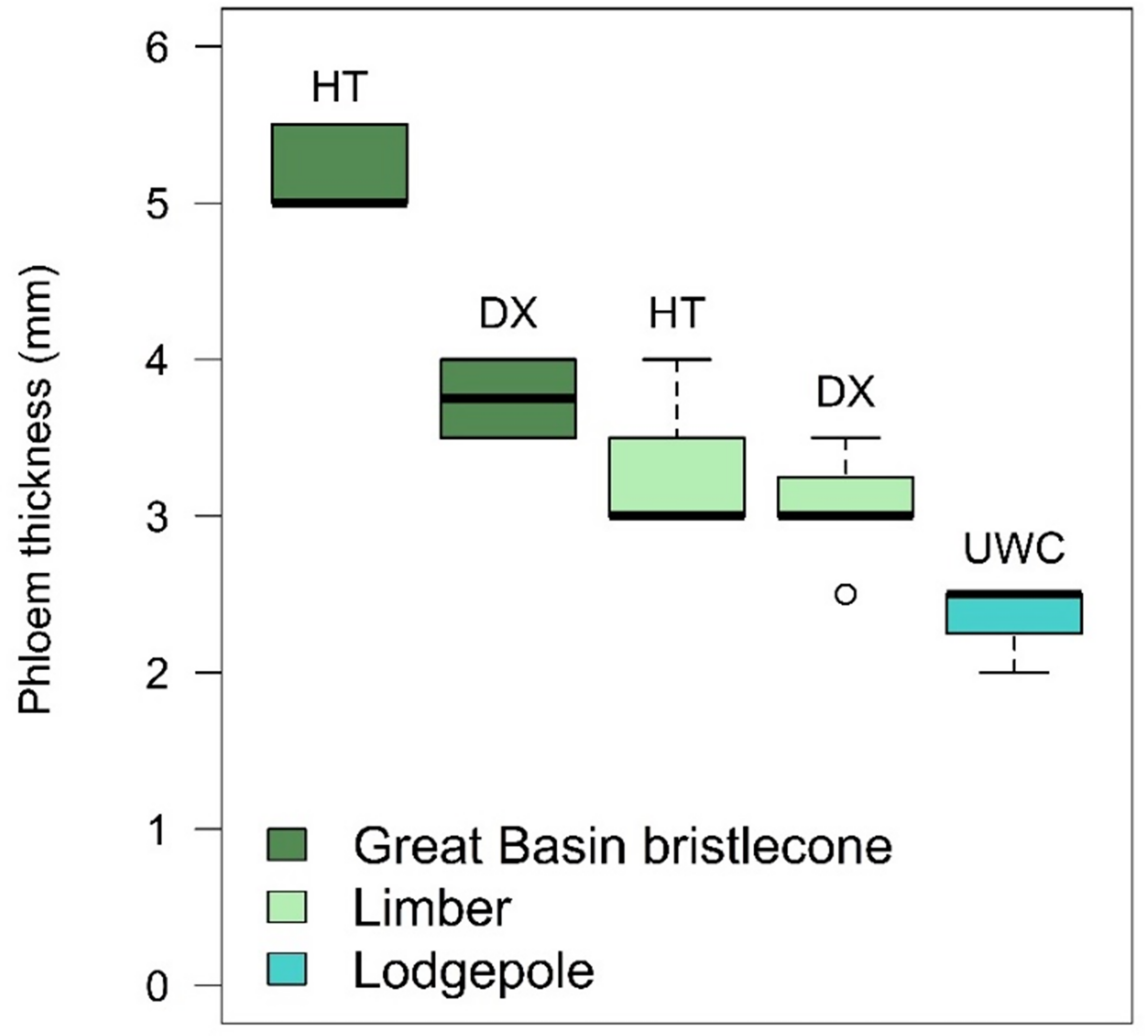
Phloem thickness of bolts cut from each of the five host trees that were manually infested with mountain pine beetle parents. HT = Humboldt-Toiyabe National Forest site, DX = Dixie National Forest site, UWC = Uinta-Wasatch-Cache National Forest site. All eight bolts from each of the Great Basin bristlecone and limber pine trees were used in phloem thickness calculations but only four of the eight lodgepole pine bolts were used due to missing data. Shown are the median (solid middle line), 25th and 75^th^ percentiles (top and bottom of box), ± 1.5 x the interquartile range (whiskers), and all outliers (points).

Although all host trees had a similar dbh of ~30–35 cm, phloem thickness differed significantly among host trees (*F*_4,31_ = 62.53, *P* < .0001). Phloem was the thickest in the GB bristlecone pines. Phloem in bristlecone BR-HT was thicker than phloem in bristlecone BR-DX (adjusted *P* < .0001), and both bristlecone pines had thicker phloem than all other host trees (adjusted *P* values range 0.0272 to < .0001 for multiple comparisons). Phloem thickness did not differ between limber pines LM-HT and LM-DX (adjusted *P* = 0.6871) and was significantly thinner in the lodgepole pine LP-UWC relative to all other host trees (adjusted *P* values range 0.0006 to < .0001 for multiple comparisons) (Fig 3).

### Mating success and fecundity

All MPB parent pairs were equally likely to mate successfully regardless of the beetle population source (*F*_1,127_ = 1.70, *P* = 0.1949) or the host tree (*F*_4,127_ = 0.90, *P* = 0.4645). The length of individual galleries constructed by successful parent MPBs by day 26 was significantly influenced by beetle population source, with beetles from the NV population constructing longer galleries than those from the UT population source (*F*_1,97_ = 17.77, *P* < 0.0001). Parent gallery length was also influenced by host tree (*F*_4,97_ = 3.43, *P* = 0.0114). Successful parent gallery lengths in bristlecone BR-DX (mean 56.4 cm for NV beetles; 36.33 cm for UT beetles) were significantly longer than in limber LM-HT (mean 38.6 cm for NV beetles; 31.0 cm for UT beetles) (adjusted *P* = 0.0187).

**Fig 4:**
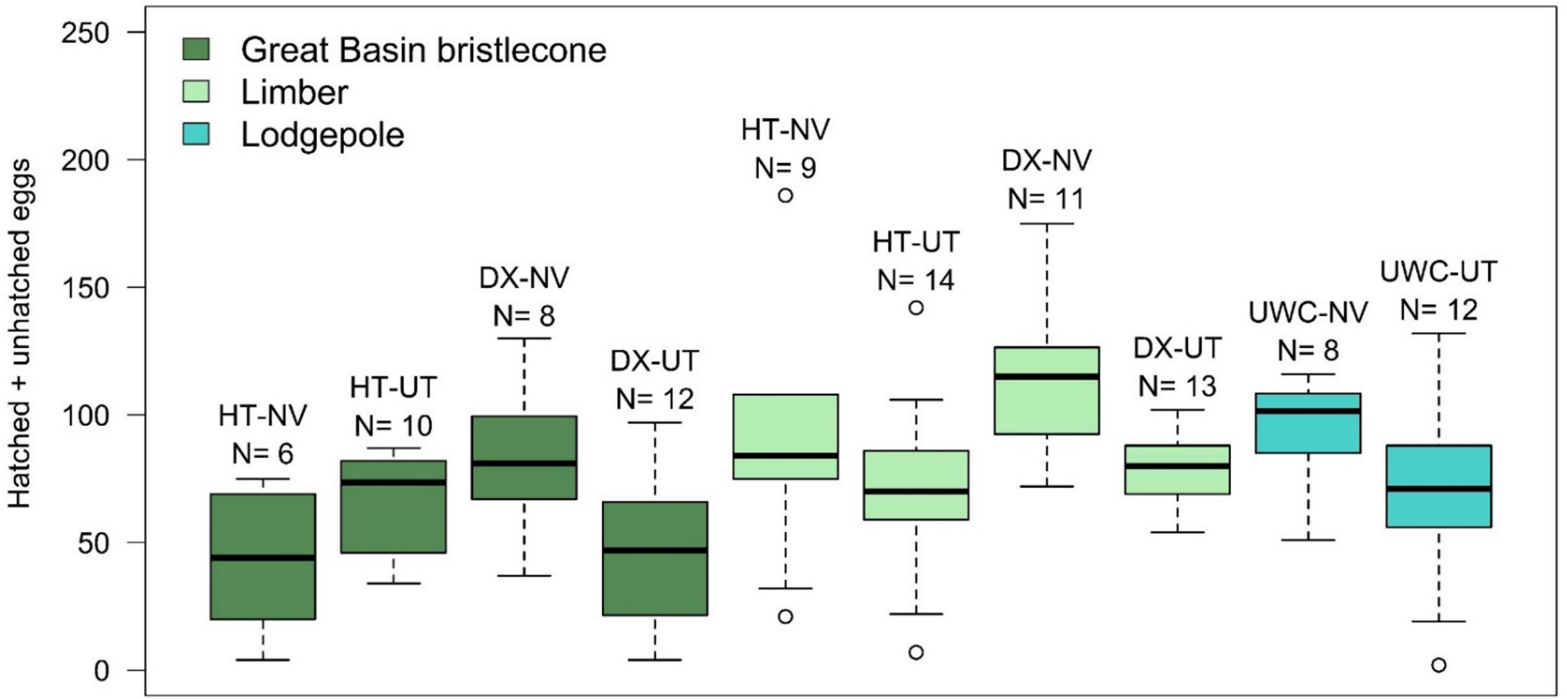
Total fecundity measured by the sum of larval galleries (hatched eggs) and eggs that had not yet hatched for each parent gallery. HT = Humboldt-Toiyabe National Forest site, DX = Dixie National Forest site, UWC = Uinta-Wasatch-Cache National Forest site; NV = Nevada beetle population, UT = Utah beetle population. Shown are the median (solid middle line), 25th and 75th percentiles (top and bottom of box), ± 1.5 x the interquartile range (whiskers), and all outliers (points). N = number of parent galleries in each category.

Total fecundity (hatched + unhatched eggs) per successful MPB parent pair was significantly influenced by beetle population source, with parent MPBs from the NV population source having higher total fecundity than parent beetles from the UT population source (*F*_1,97_ = 6.19, *P* = 0.0146) (Fig 4). Total fecundity was also influenced by host tree (*F*_4,97_ = 2.78, *P* = 0.0310). The total fecundity of MPB parents placed in limber LM-DX was significantly higher than that of parent beetles placed in bristlecone BR-DX (adjusted *P* = 0.0422) (Fig 4).

### Offspring emergence

**Fig 5:**
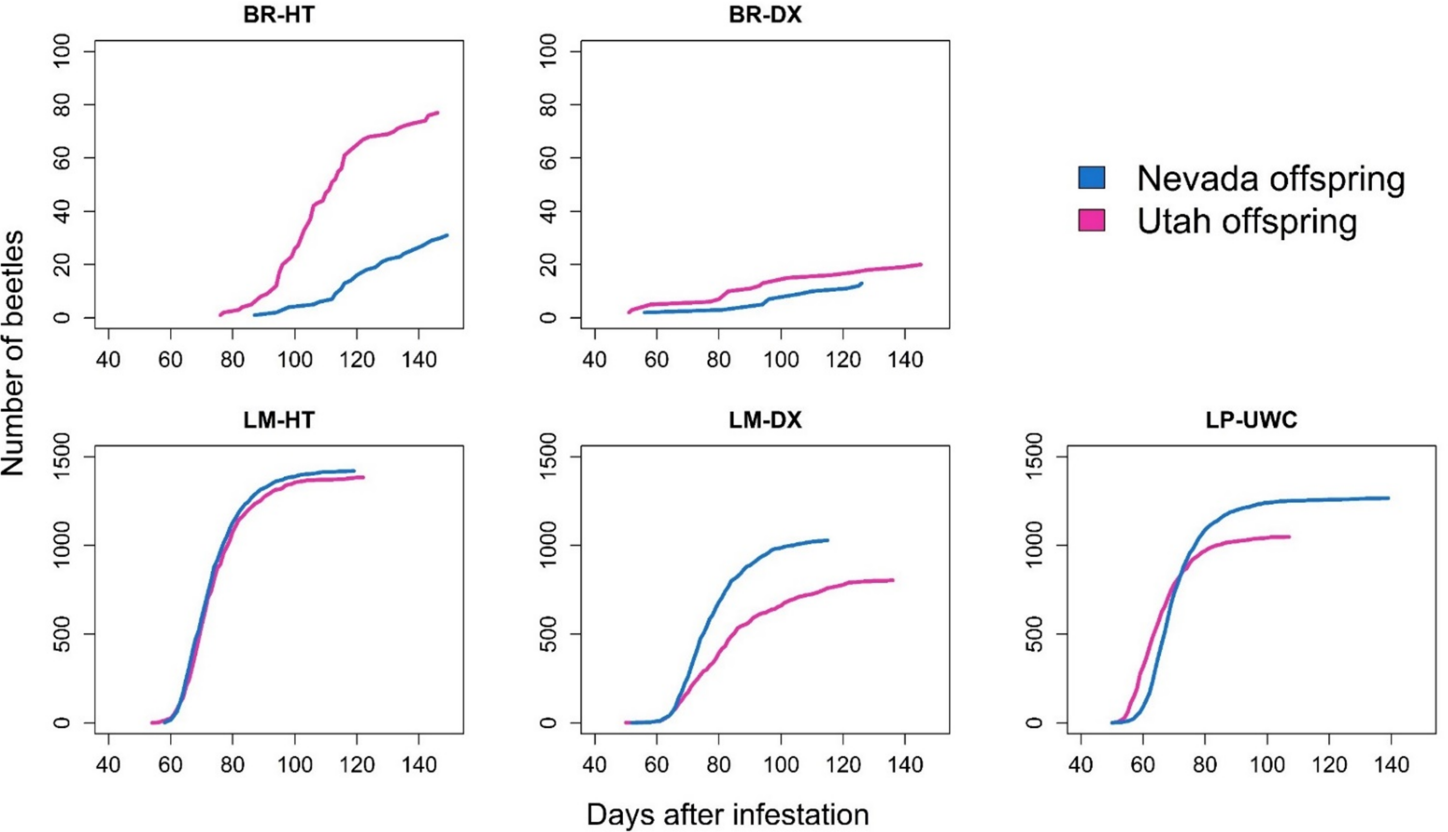
Cumulative total numbers of emerged offspring by host tree and mountain pine beetle population for the entire emergence period, unadjusted for slightly differing numbers of successful parent galleries in each host tree. BR = Great Basin bristlecone pine, LM = limber pine, LP = lodgepole pine; HT = Humboldt-Toiyabe National Forest site, DX = Dixie National Forest site, UWC = Uinta-Wasatch-Cache National Forest site. Emerging offspring were collected from six individually infested bolts of each host tree starting at day 50 through day 100 after infestation and until either five consecutive days had passed with no newly emerged offspring from the bolt or until day 150 was reached. Note Y axis limit is only 100 for Great Basin bristlecone pine graphs but Y axis limit is 1,500 for graphs for other tree species.

Extremely few total offspring emerged from GB bristlecone pine host trees (108 total from BR-HT; 33 total from BR-DX) relative to the other host tree species (2,802 total from LM-HT; 1,833 from LM-DX; 2,314 from LP-UWC) (Fig 5). Degradation of the phloem and the deceased offspring over the course of the experiment obscured individuals and their larval galleries, so we were not able to quantify percent brood mortality in any of the host trees. However, post-emergence peeling of the phloem revealed evidence of many dead offspring in the bristlecone bolts, indicating most offspring died prior to completing development.

**Fig 6:**
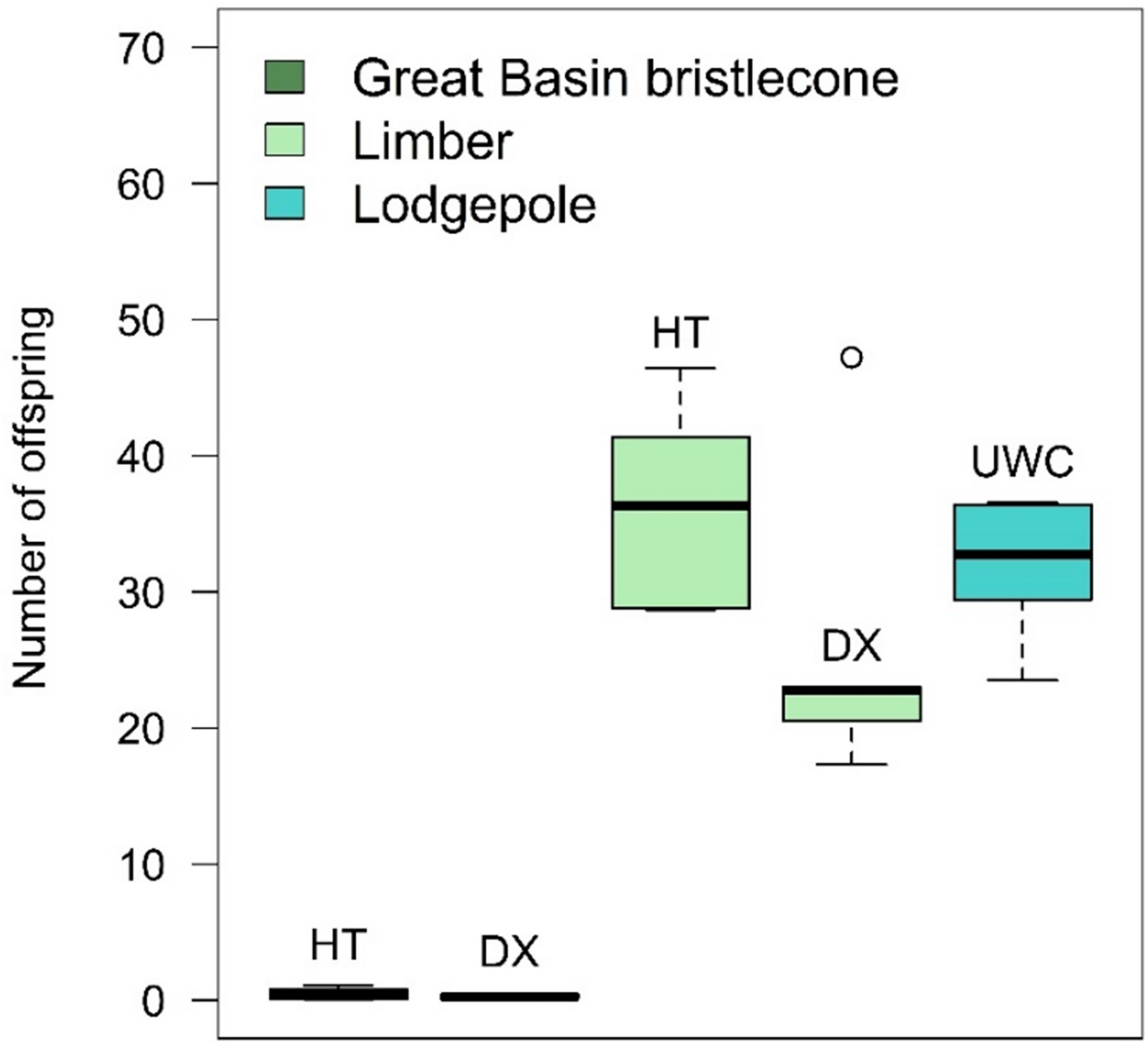
Average total number of emerged offspring per successful parent gallery by host tree for days 50 through 100 after infestation. HT = Humboldt-Toiyabe National Forest site, DX = Dixie National Forest site, UWC = Uinta-Wasatch-Cache National Forest site. In both Great Basin bristlecone pines, the average total number of offspring per successful parent pair was <1. There were six bolts of each host tree. Results from Utah and Nevada beetles are combined because there was no significant difference in average total number of emerged offspring per successful parent gallery between mountain pine beetle populations. Shown are the median (solid middle line), 25th and 75th percentiles (top and bottom of box), ± 1.5 x the interquartile range (whiskers), and all outliers (points).

When adjusting for the number of successful parent galleries in each bolt and standardizing the offspring emergence period to days 50–100 after infestation, the average total number of emerged offspring per successful parent gallery was strongly influenced by host tree (*F*_4,24_ = 175.94, *P* < .0001), with bolts from the GB bristlecone pine trees producing significantly fewer emerged offspring than the other host trees. The average total number of offspring per successful parent pair was <1 for both GB bristlecone pines (Fig 6), meaning that the number of offspring that emerged from GB bristlecone pine was fewer than the number of parent beetles that were manually inserted into this species. There were no significant differences in the average total number of emerged offspring per successful parent gallery between the limber and lodgepole pine host trees (adjusted *P* values range 0.0852 to 0.9021 for multiple comparisons). Average total offspring emergence per successful parent gallery was not influenced by the MPB parental population (*F*_1,24_ = 0.06, *P* = 0.8124).

Similar to results from the 26-day mating and fecundity data, successful parent gallery length in bolts held at 22.5°C for the entire emergence period was significantly greater for the NV beetles relative to the UT beetles (*F*_1,24_ = 9.46, *P* = 0.0052). Also similar to mating and fecundity data, successful parent gallery length was influenced by host tree (*F*_4,24_ = 4.23, *P* = 0.0099). Again, successful parent galleries in bristlecone BR-DX (NV mean = 44.3 cm; UT mean = 46.3 cm) were significantly longer than those constructed in limber LM-HT (NV mean = 38.7 cm; UT mean = 30.7 cm) (adjusted *P* = 0.0038).

**Fig 7:**
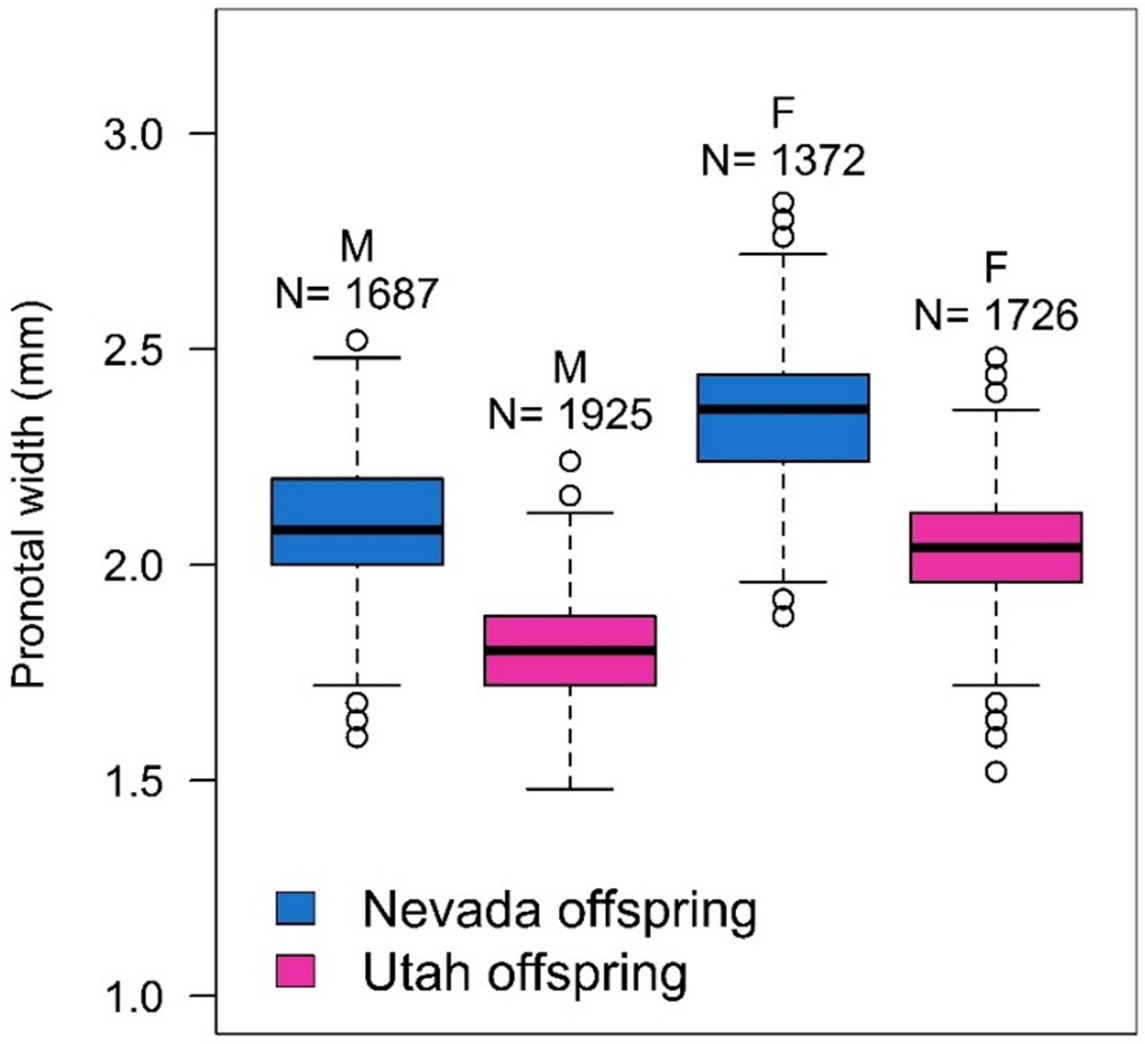
Male (M) and female (F) offspring mountain pine beetle size from the Nevada and Utah populations. To standardize the emergence period across all bolts, only mountain pine beetles that emerged days 50–100 were used in size calculations. Shown are the median (solid middle line), 25th and 75th percentiles (top and bottom of box), ± 1.5 x the interquartile range (whiskers), and all outliers (points). N = number of beetles in each category.

**Fig 8:**
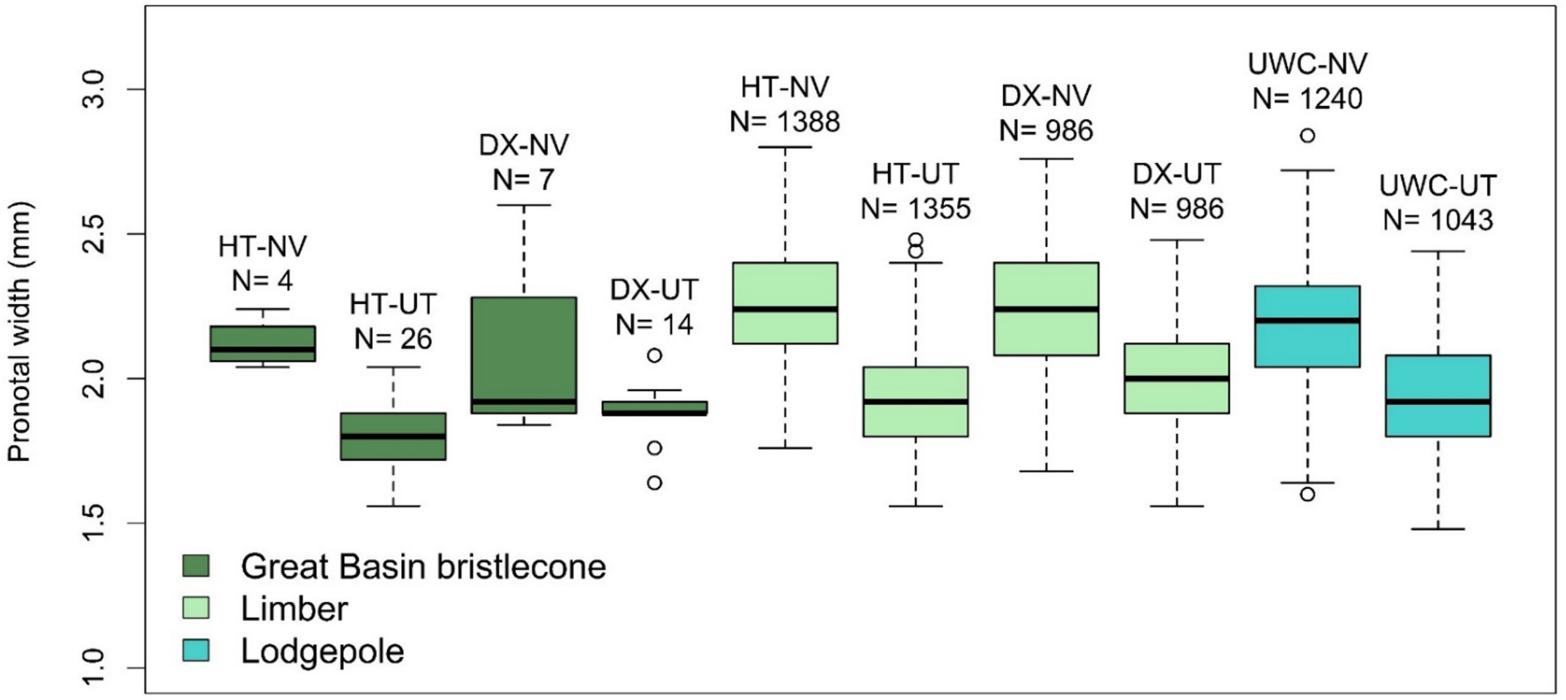
Female offspring size by host tree and mountain pine beetle population. HT = Humboldt-Toiyabe National Forest site, DX = Dixie National Forest site, UWC = Uinta- Wasatch-Cache National Forest site; NV = Nevada beetle population, UT = Utah beetle population. Female offspring from the Great Basin bristlecone pines were significantly smaller than those from the limber pines, and females from HT Great Basin bristlecone pine were significantly smaller than females from lodgepole pine. Male offspring size was not significantly different between host trees. Shown are the median (solid middle line), 25^th^ and 75th percentiles (top and bottom of box), ± 1.5 x the interquartile range (whiskers), and all outliers (points). N = number of beetles in each category.

Both male and female offspring from the NV MPB population were larger than those from the UT population source (males: *F*_1,20_ = 113.33, *P* < .0001; females: *F*_1,22_ = 152.16, *P* < .0001) (Fig 7). Host tree significantly affected female offspring size (*F*_4,22_ = 7.10, *P* = 0.0008) but not male offspring size (*F*_4,20_ = 2.41, *P* = 0.0828). Female offspring from both GB bristlecone pines were smaller than those from limber pine (adjusted *P* values range 0.0296 to 0.0031 for multiple comparisons), and females from bristlecone BR-HT were also smaller than females from lodgepole pine (adjusted *P* = 0.0453) (Fig 8).

## Discussion

Our findings in laboratory tests demonstrate that GB bristlecone pine is a poor host for MPB offspring. Together with previous field studies demonstrating low MPB preference for GB bristlecone pine [22,24], these findings are consistent with a low oviposition preference—low offspring performance relationship. Therefore, low MPB preference for GB bristlecone pine can be considered an adaptive trait, and is expected to be maintained unless other adaptations evolve which improve the low offspring performance.

Despite mating and oviposition success in all three tree species in our study, extremely few offspring survived to complete development in GB bristlecone pine relative to lodgepole and limber pines. Furthermore, offspring that did survive in GB bristlecone pine were less fit than those from the other host tree species: beetles emerged asynchronously and females were smaller in size. Asynchronous emergence hinders the ability of MPBs to maintain seasonality and carry out mass-attacks on new host trees [39], and small female size is hypothesized to reflect lower energy reserves for host-searching [40] and lower survival when exposed to host toxins [41]. Although parent sizes for each beetle population did differ across some host trees, the small size of GB bristlecone-reared female offspring is likely a host effect rather than an inherited trait. Parent beetles infested into GB bristlecone pine were not consistently smaller than those infested into other host trees, and parent size differences among limber and lodgepole pine did not translate to differences in offspring size.

Phloem thickness, regardless of host tree species, has often been considered the most important factor in MPB brood production [28,38]. In our study, however, GB bristlecone pine had the thickest phloem and produced the fewest offspring, and lodgepole pine had the thinnest phloem yet produced as many, similarly-sized offspring as limber pines with relatively thick phloem. While phloem quantity may be an important predictor of MPB offspring performance when host defenses are similar, phloem quality (i.e., chemical makeup) may be a more important determinant of MPB offspring production when comparing host tree species with varying defense levels. The effects of the high concentrations of constitutive toxins in GB bristlecone pine phloem [22] may outweigh the benefit of high phloem abundance for developing MPB larvae.

We did not find evidence that the NV beetles, which were collected from a limber pine within GB bristlecone pine range, performed better in GB bristlecone than the UT beetles, which were collected from a lodgepole pine outside of GB bristlecone pine range. Although the NV parent beetles were larger and more fecund than the UT parent beetles, offspring emergence was not higher for the NV beetles relative to the UT beetles in any of the tree species. Similar to their parents, NV offspring were larger in size than UT offspring, and their size combined with initially higher numbers of larvae may have led to higher mortality due to intraspecific competition. Furthermore, the hypothesized superior detoxification abilities of MPBs raised in limber pine (NV parents) as compared to lodgepole pine (UT parents) [27] evidently did not affect offspring survival in GB bristlecone pine. In fact, although there were no significant differences between the population sources in the average number of offspring per successful parent pair in GB bristlecone pine, raw total numbers of offspring were lower for NV beetles relative to UT beetles in GB bristlecone pine (Fig 5).

Why did MPB offspring develop successfully in lodgepole and limber pine, but not in GB bristlecone pine? A number of factors could lead to poor MPB offspring performance in a given host tree species, but our study focused specifically on the effects of the constitutive phloem environment. We simulated successful colonization through manual infestation of parent beetles, we removed the capacity for induced host tree defenses by cutting host trees prior to infestation, and we controlled for competition and external effects in laboratory experiments. Host tree bolts presumably retained many constitutive qualities, including nutrients, physical traits, and pre-formed chemical compounds. Given these circumstances, several hypotheses may explain poor offspring performance in GB bristlecone pine phloem.

One hypothesis to explain poor MPB offspring survival in GB bristlecone pine is insufficient available nutrition, either because the phloem is nutrient-poor, or because the establishment of mutualistic fungi and bacteria associated with MPB is inhibited. Low availability of phloem nutrients such as nitrogen can limit insect development, and microbial symbionts, including mutualistic ‘blue-stain’ fungi, play an important role in bark beetle nutrient acquisition [42,43]. Although we did not directly test for the presence of microbial symbionts, we did observe blue staining in the sapwood of lab-infested GB bristlecone pine, which is associated with MPB mutualistic fungi. Long parent galleries are hypothesized to reflect low-quality phloem because a greater quantity of phloem is needed to meet nutritional needs [27]. In our study, GB bristlecone pine BR-DX produced the fewest offspring and had significantly longer parent galleries than limber LM-HT, which produced the most offspring. This difference may support the *insufficient nutrients* hypothesis. However, although offspring performance in bristlecone BR-HT was also very poor, parent galleries in BR-HT were not significantly longer than galleries in the other host trees, so our evidence is inconclusive. Furthermore, insufficient nutritional resources in GB bristlecone pine phloem would be surprising because phloem thickness was greatest in GB bristlecone pine, and therefore was available in the highest abundance.

A second hypothesis is that GB bristlecone pine possesses constitutive phloem compounds and defenses that inhibit developing MPB offspring. Bentz et al. (2016) [22] found that GB bristlecone pine has over eight times the concentration of constitutive phloem compounds found in limber pine, and many of these compounds have been associated with high toxicity and tree defense [31]. The Resource Availability Hypothesis predicts that slow-growing plants will invest heavily in constitutive anti-herbivore defenses due to the high cost of replacing tissue [44]. GB bristlecone pine, a slow-growing tree, is therefore expected to invest heavily in constitutive defenses that negatively affect phytophagous insects. We found that mating and oviposition of viable eggs occurred in GB bristlecone pine, but that the majority of individuals died prior to emergence. Phloem toxins would be expected to have a greater effect on developing offspring as compared to reproducing adults due to the longer exposure time required for larvae to develop relative to the shorter time required for adults to mate and oviposit.

If high concentrations of constitutive phloem toxins are responsible for low MPB offspring performance in GB bristlecone pine, why aren’t these same defense traits present in co-occurring limber pine, which can also be considered a slow-growing species? Constitutive defenses in pines are closely linked with their macroevolutionary history, and the absence of shared selective pressures over long time scales can result in differences in plant defenses [45]. Tree species in the bristlecone pine group, including the ancestors of modern GB bristlecone pine, have been growing in the western United States for at least 40 million years [25]. Although it is possible that limber pine was also present in this area millions of years ago, the oldest limber pine fossils to date are fewer than 50,000 years old [46]. It is difficult to infer which selective agents may have interacted with these tree species over their long evolutionary histories, but perhaps longer or more intense past herbivore pressure in GB bristlecone pine gave rise to a unique arsenal of anti-herbivory defenses against phytophagous insects. Furthermore, the greater longevity in GB bristlecone pine relative to limber pine may aid in the maintenance of highly effective defense traits over time despite interrupted contact with selective phytophagous agents [47].

Low MPB preference for GB bristlecone pine is well documented [22–24] and our results show that most MPB offspring die before completing development in GB bristlecone pine. The high MPB offspring mortality in GB bristlecone pine is unique among pine species. All other pine species that have been tested for MPB offspring performance, including those found outside of the current MPB range, have been shown to be suitable for developing offspring, at least when cut [48–50]. Studies have also shown that MPBs can successfully propagate in some species of spruce (*Picea*) [48,51], which is not generally considered to be a MPB host genus. Jeffrey pine (*P*. *jeffreyi*), which like GB bristlecone pine is not attacked by MPB in nature, has been shown to be a more favorable host for MPB offspring performance than ponderosa pine (*P*. *ponderosae*) and sugar pine (*P*. *lambertiana*) [50], both of which are readily attacked. The low oviposition preference yet high performance in Jeffrey pine is in contrast to the strongly correlated low oviposition preference-low offspring performance relationship between MPB and GB bristlecone pine. Evidence suggests that MPB and Jeffrey pine beetle (*D*. *jeffreyi*), a MPB relative that exclusively attacks Jeffrey pine, diverged between 2 and 8 million years ago [52] and that Jeffrey pine beetle was the first of the two species to arrive in Jeffrey pine range [50]. If MPB’s low preference-high performance relationship with Jeffrey pine is an example of insufficient time for oviposition preferences to adapt to a suitable host [5], perhaps the well-adapted, strongly correlated low preference-low performance in GB bristlecone pine is indicative of a longer evolutionary association, beginning >2–8 million years ago.

## Conclusions

The number of MPB offspring that emerged from GB bristlecone pine was less than the number of parent beetles that successfully mated in this species, indicating strong selective pressure to maintain low MPB preference for GB bristlecone pine. Our laboratory results using trees from two sites within GB bristlecone pine range support previous findings of low MPB preference for GB bristlecone growing across the extent of its range [22]. Additional research including a greater number of trees growing across GB bristlecone pine range will clarify whether variation in host suitability for MPB reproduction occurs. Understanding specific tree traits responsible for the death of developing offspring, in addition to potential barriers to MPB colonization (i.e., the potential for induced defenses and constitutive bark defenses), will facilitate the development of new tools to protect forest communities susceptible to MPB outbreaks and increase understanding of the evolution of anti-herbivore defenses. Although the evolutionary history between MPB and GB bristlecone pine is unclear, a low preference-low performance relationship suggests that Great Basin bristlecone pine resistance to mountain pine beetle is likely to be retained through climate-driven high-elevation mountain pine beetle outbreaks.

## Supporting information

S1 Fig Photos of manually infesting cut tree bolts with mountain pine beetle parent pairs. Panel (a): We initiated ~2 cm deep parent galleries with a drill at equal spacing along the circumference of a bolt. Panel (b): We manually inserted parent beetles into galleries and covered gallery openings with screen. Panel (c): The bottom of a fully infested bolt.
(JPG)

S2 Fig Photo of infested tree bolt caged in screen to facilitate daily collections of emerged offspring. Infested bolts were held in incubator cabinets at 22.5°C and emerging offspring were collected from day 50 up to day 150 following infestation.
(JPG)

S1 Supporting Information Residual plots for each statistical model used in analyses. Plots were generated using SAS Studio version 9.4.
(PDF)

S1 Dataset All data files used in analyses. The dataset consists of a zipped folder containing 6 .csv files and a metadata text file.
(ZIP)
