# Supplementary figures and images for: Low offspring survival in mountain pine beetle infesting the resistant Great Basin bristlecone pine supports the preference-performance hypothesis
Source: PLoS One. 2018 May 1;13(5):e0196732. doi: 10.1371/journal.pone.0196732 (PMC5929522; doi:10.1371/journal.pone.0196732)

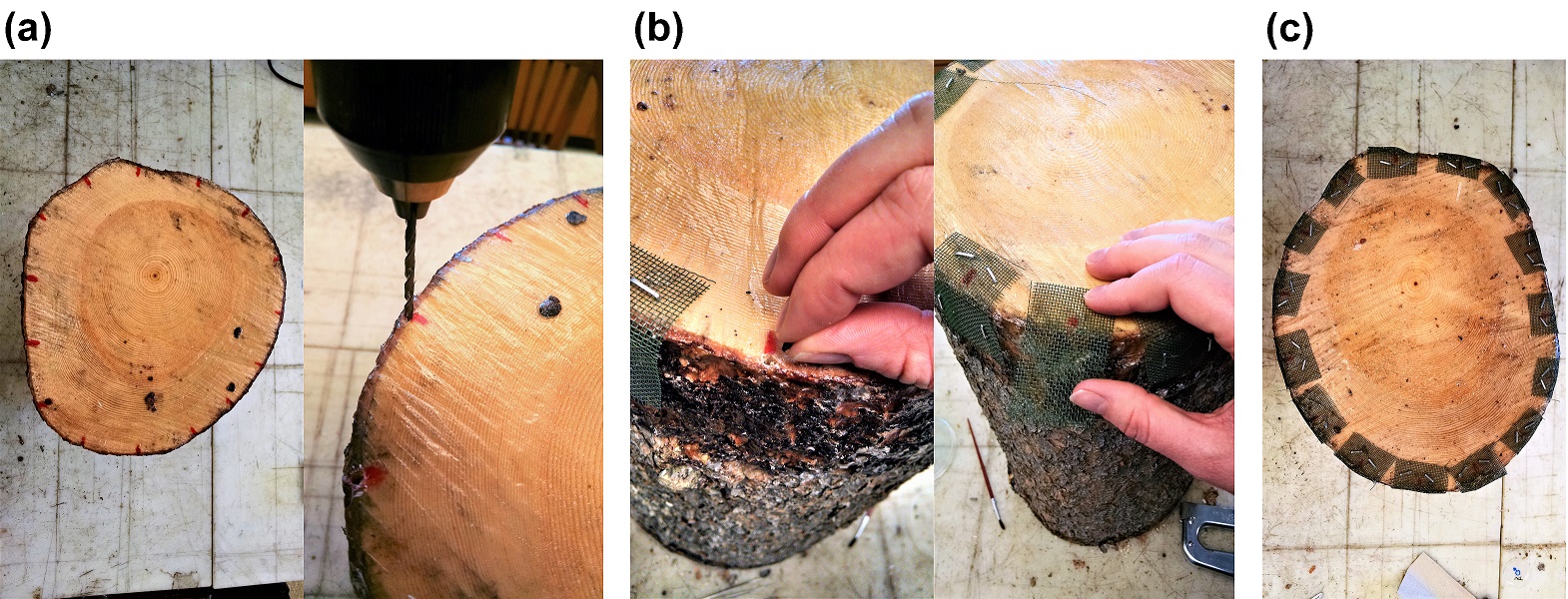

Supplement: S1 Fig — Panel (a): We initiated ~2 cm deep parent galleries with a drill at equal spacing along the circumference of a bolt. Panel (b): We manually inserted parent beetles into galleries and covered gallery openings with screen. Panel (c): The bottom of a fully infested bolt. (JPG) [file pone.0196732.s001.jpg]

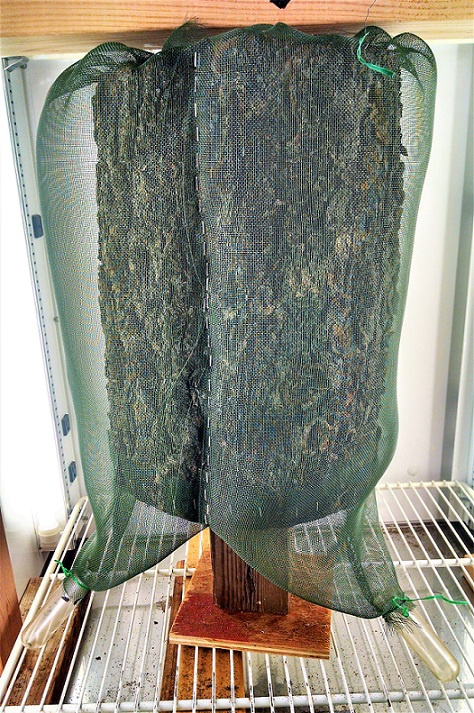

Supplement: S2 Fig — Infested bolts were held in incubator cabinets at 22.5°C and emerging offspring were collected from day 50 up to day 150 following infestation. (JPG) [file pone.0196732.s002.jpg]
