# Supplementary material for: Low offspring survival in mountain pine beetle infesting the resistant Great Basin bristlecone pine supports the preference-performance hypothesis
Source: PLoS One. 2018 May 1;13(5):e0196732. doi: 10.1371/journal.pone.0196732 (PMC5929522; doi:10.1371/journal.pone.0196732)

Male parent size

The GLIMMIX Procedure

Model Information	
Data Set	WORK.PSIZE
Response Variable	M_Parent_Size
Response Distribution	Gaussian
Link Function	Identity
Variance Function	Default
Variance Matrix	Diagonal
Estimation Technique	Maximum Likelihood
Degrees of Freedom Method	Residual

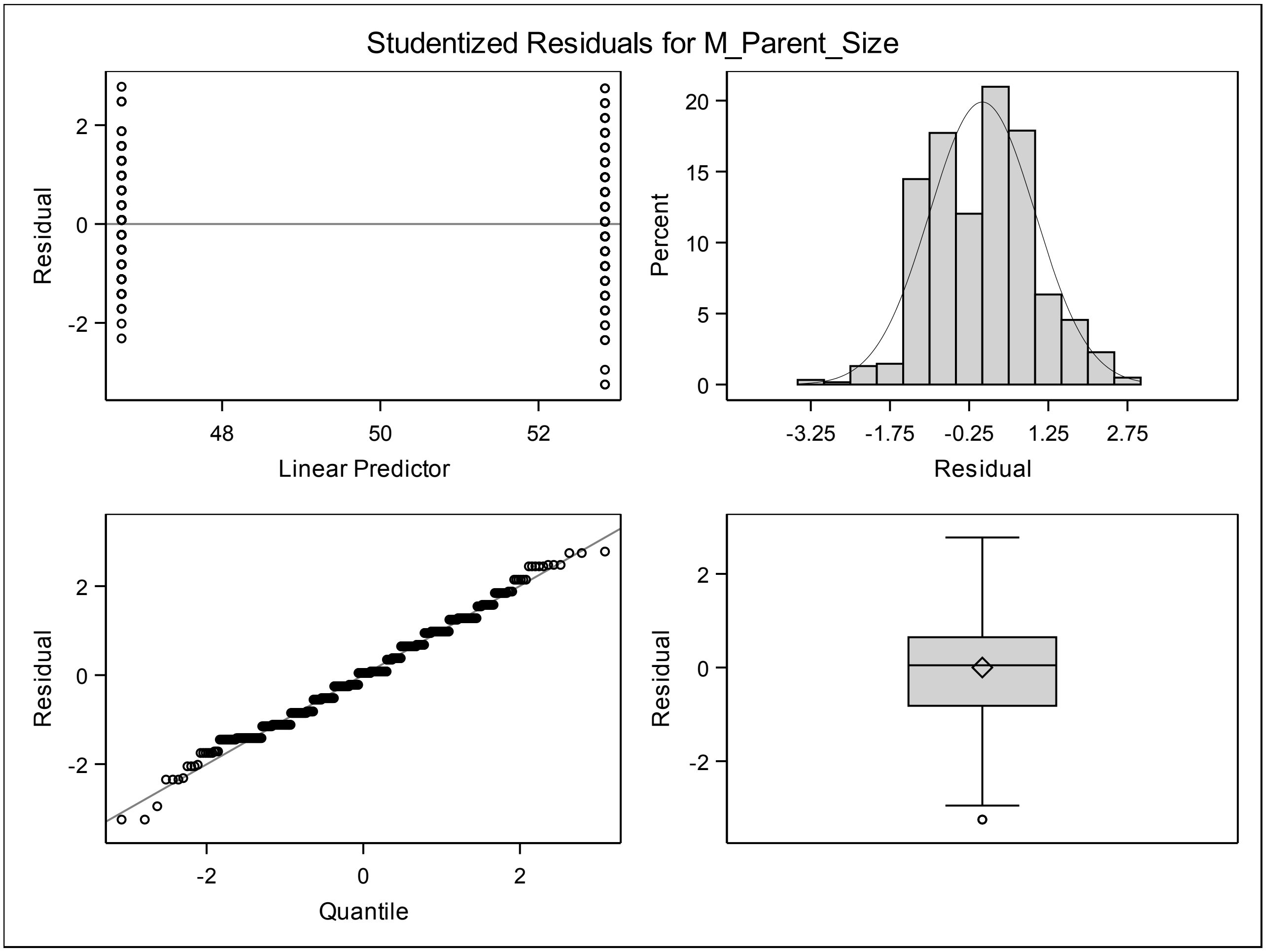

Female parent size

The GLIMMIX Procedure

Model Information	
Data Set	WORK.PSIZE
Response Variable	F_Parent_Size
Response Distribution	Gaussian
Link Function	Identity
Variance Function	Default
Variance Matrix	Diagonal
Estimation Technique	Maximum Likelihood
Degrees of Freedom Method	Residual

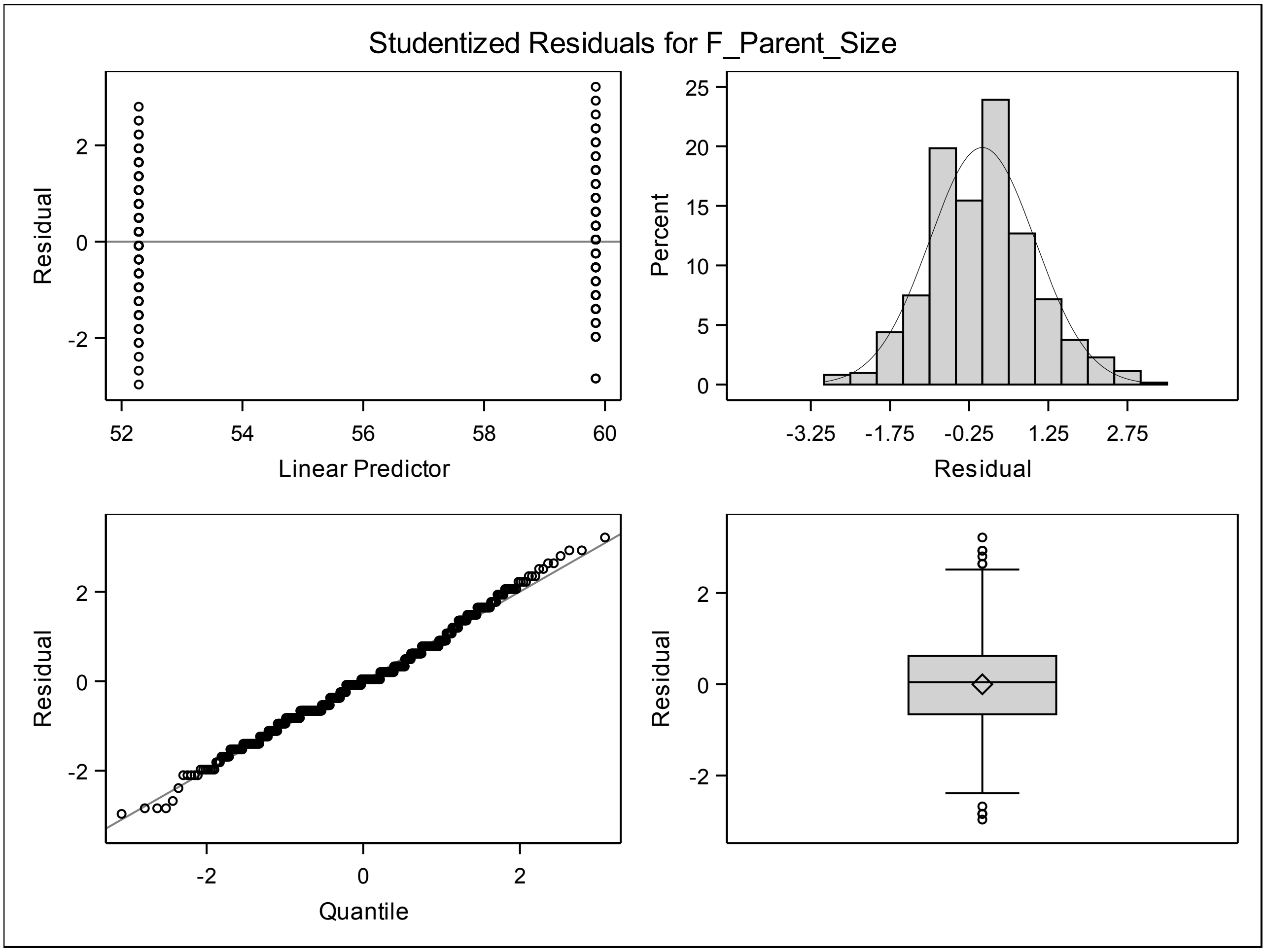

Male parent size NV

The GLIMMIX Procedure

Model Information	
Data Set	WORK.PSIZE
Response Variable	M_Parent_Size
Response Distribution	Gaussian
Link Function	Identity
Variance Function	Default
Variance Matrix	Diagonal
Estimation Technique	Maximum Likelihood
Degrees of Freedom Method	Residual

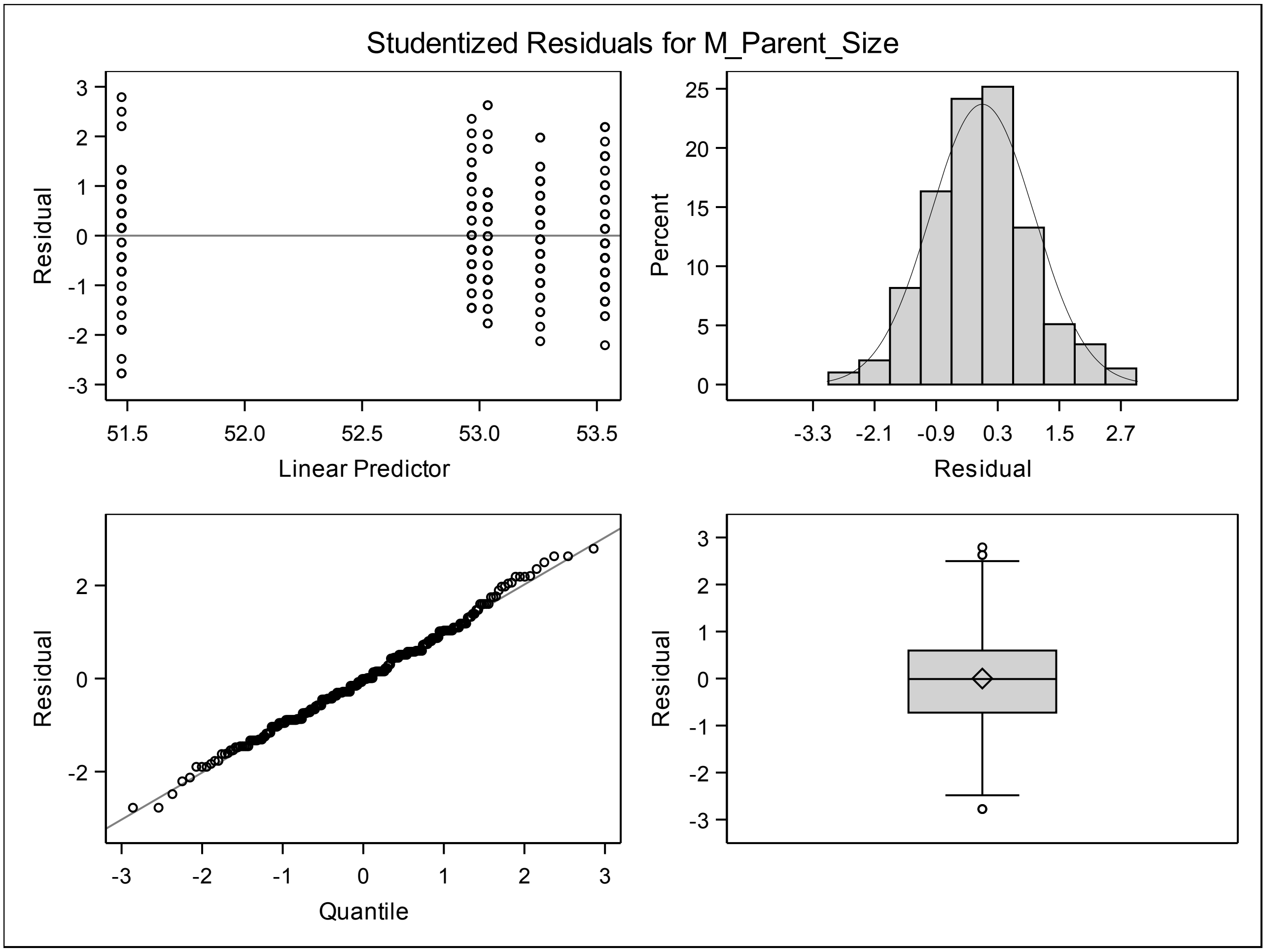

Female parent size NV

The GLIMMIX Procedure

Model Information	
Data Set	WORK.PSIZE
Response Variable	F_Parent_Size
Response Distribution	Gaussian
Link Function	Identity
Variance Function	Default
Variance Matrix	Diagonal
Estimation Technique	Maximum Likelihood
Degrees of Freedom Method	Residual

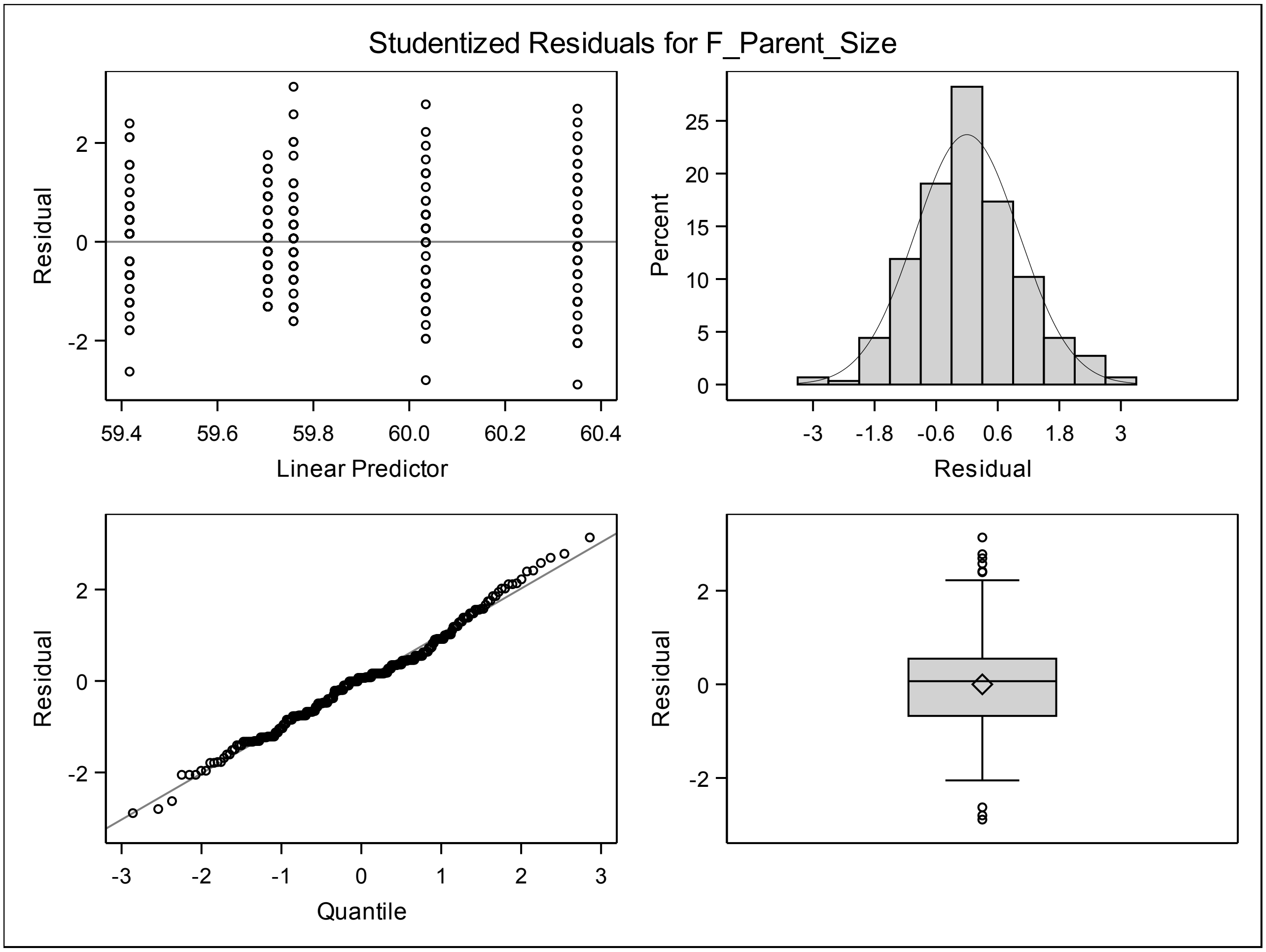

Male parent size UT

The GLIMMIX Procedure

Model Information	
Data Set	WORK.PSIZE
Response Variable	M_Parent_Size
Response Distribution	Gaussian
Link Function	Identity
Variance Function	Default
Variance Matrix	Diagonal
Estimation Technique	Maximum Likelihood
Degrees of Freedom Method	Residual

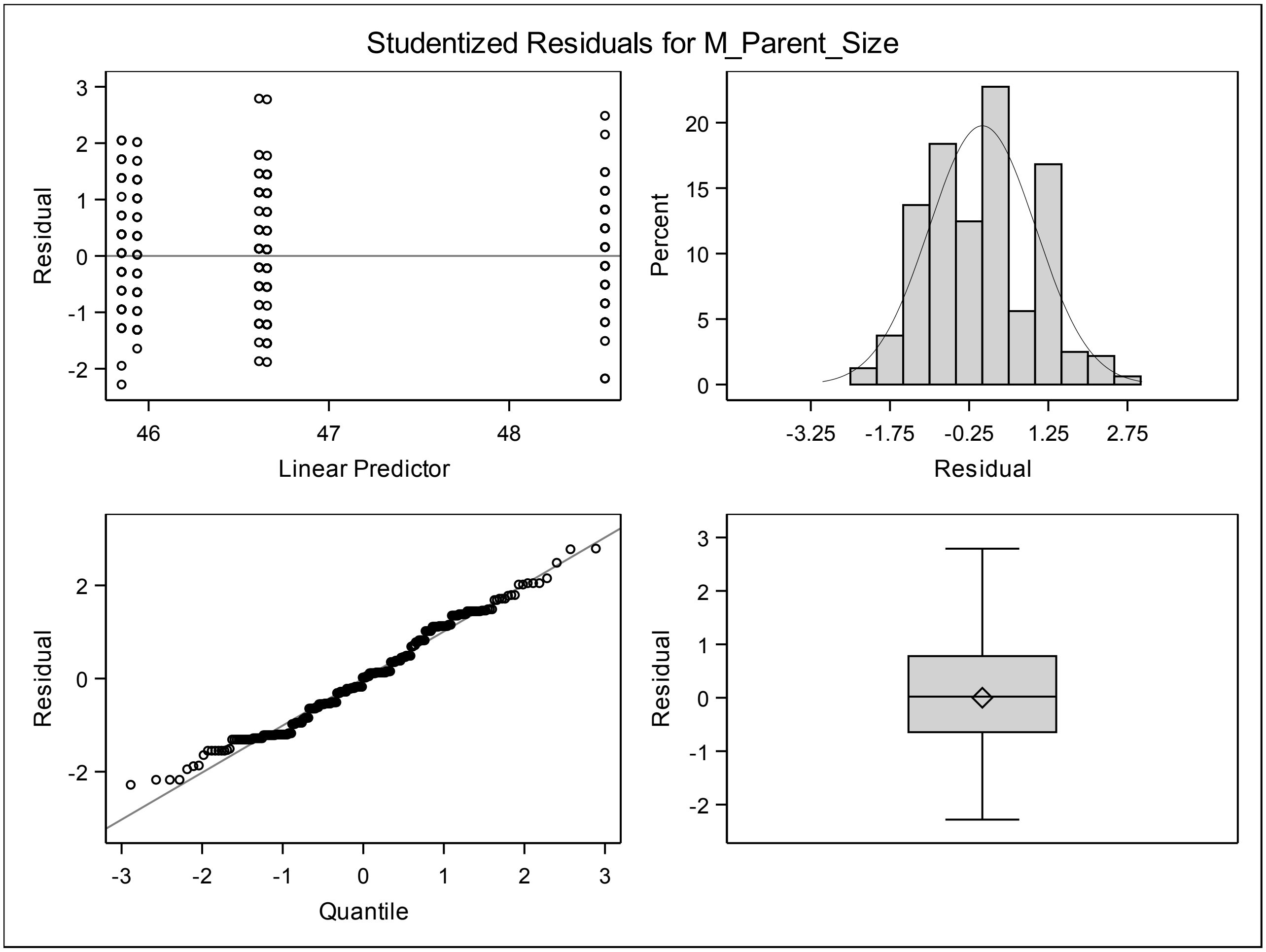

Female parent size UT

The GLIMMIX Procedure

Model Information	
Data Set	WORK.PSIZE
Response Variable	F_Parent_Size
Response Distribution	Gaussian
Link Function	Identity
Variance Function	Default
Variance Matrix	Diagonal
Estimation Technique	Maximum Likelihood
Degrees of Freedom Method	Residual

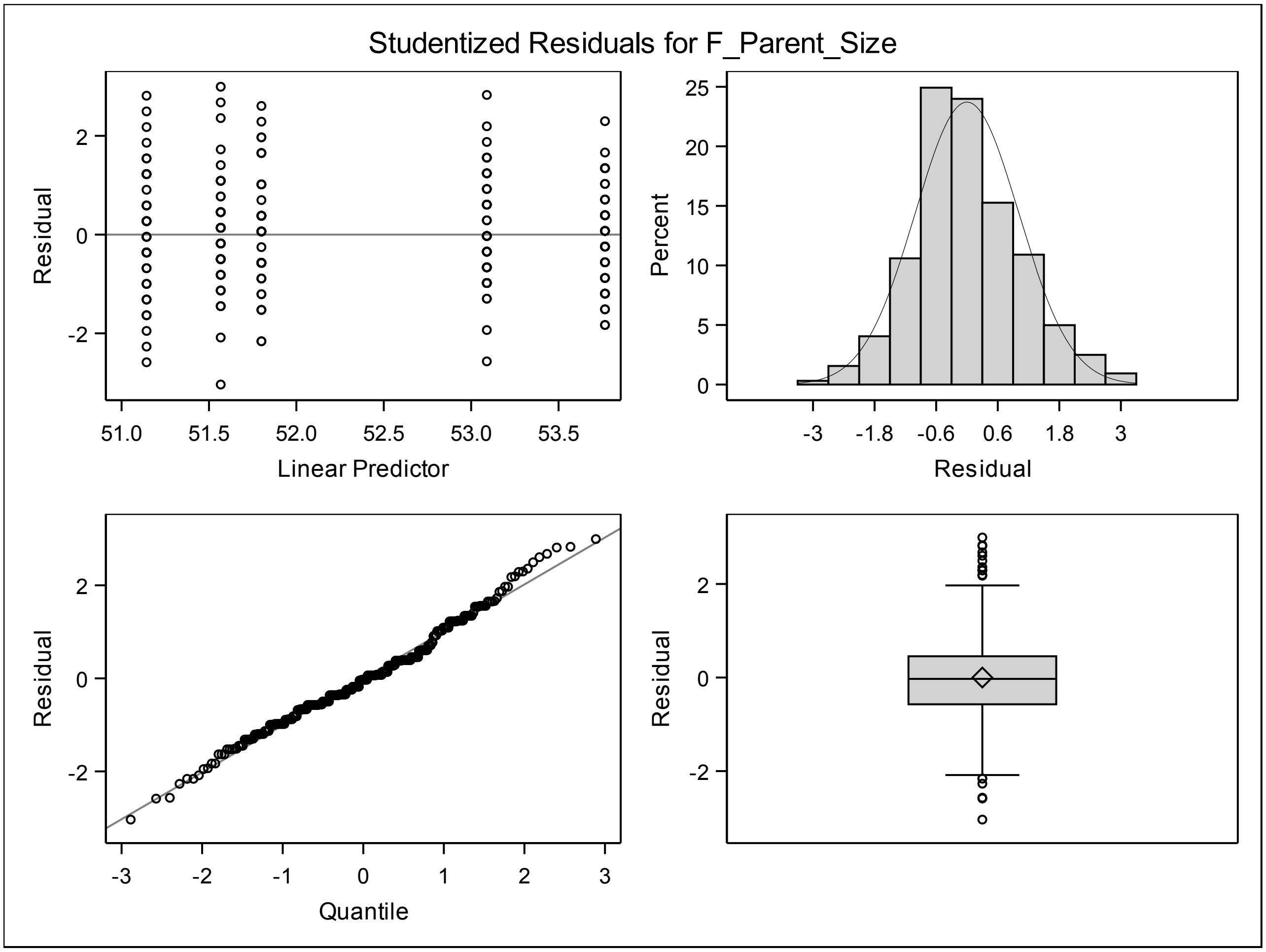

Phloem Thickness

The GLIMMIX Procedure

Model Information	
Data Set	WORK.PHLOEM
Response Variable	Phloem_Thickness_mm
Response Distribution	Gamma
Link Function	Log
Variance Function	Default
Variance Matrix	Diagonal
Estimation Technique	Maximum Likelihood
Degrees of Freedom Method	Residual

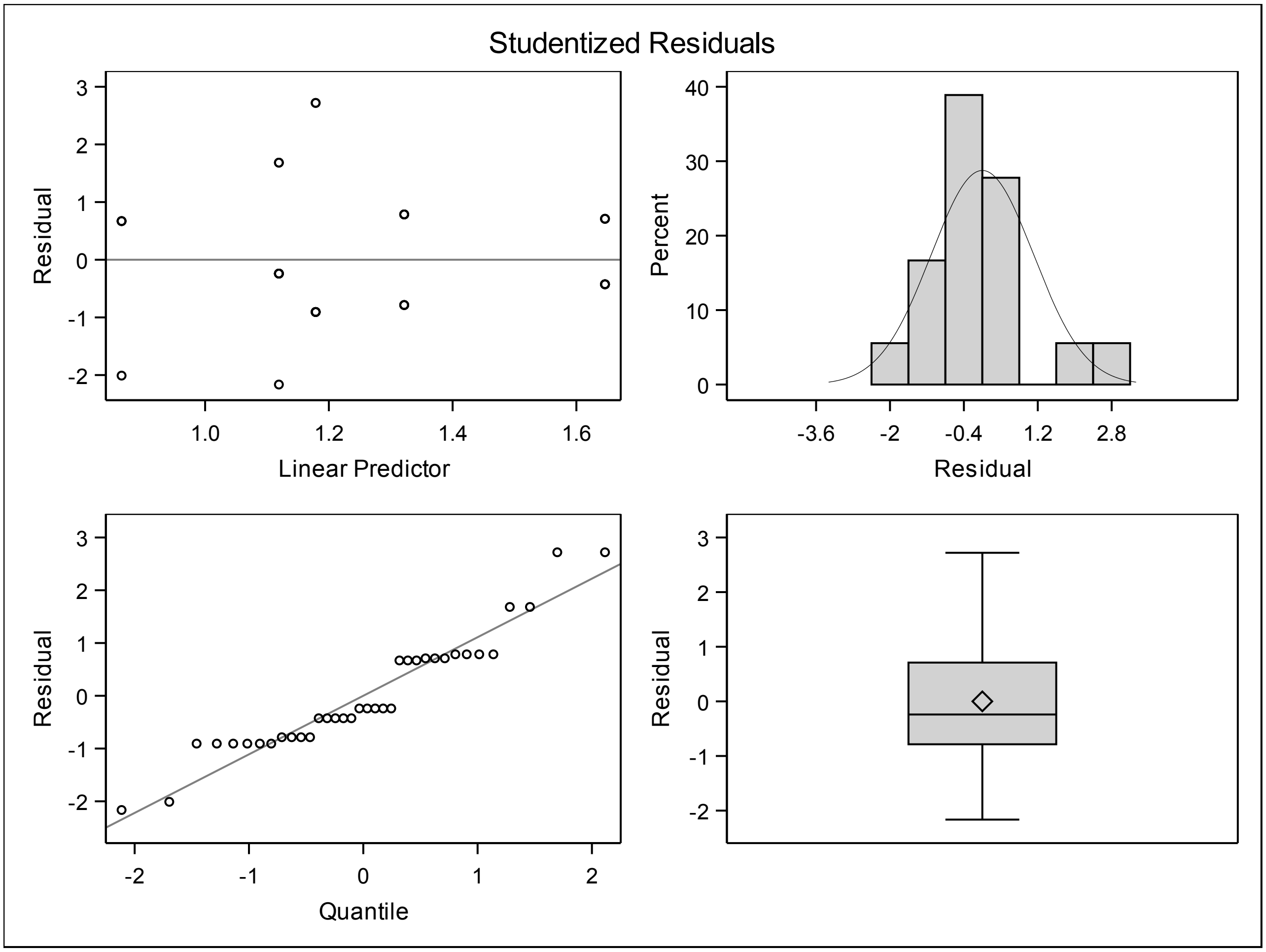

Mating and Fecundity- Probability of parent gallery success

The GLIMMIX Procedure

Model Information	
Data Set	WORK.MF
Response Variable	Gallery_successful
Response Distribution	Binary
Link Function	Logit
Variance Function	Default
Variance Matrix	Diagonal
Estimation Technique	Maximum Likelihood
Degrees of Freedom Method	Residual

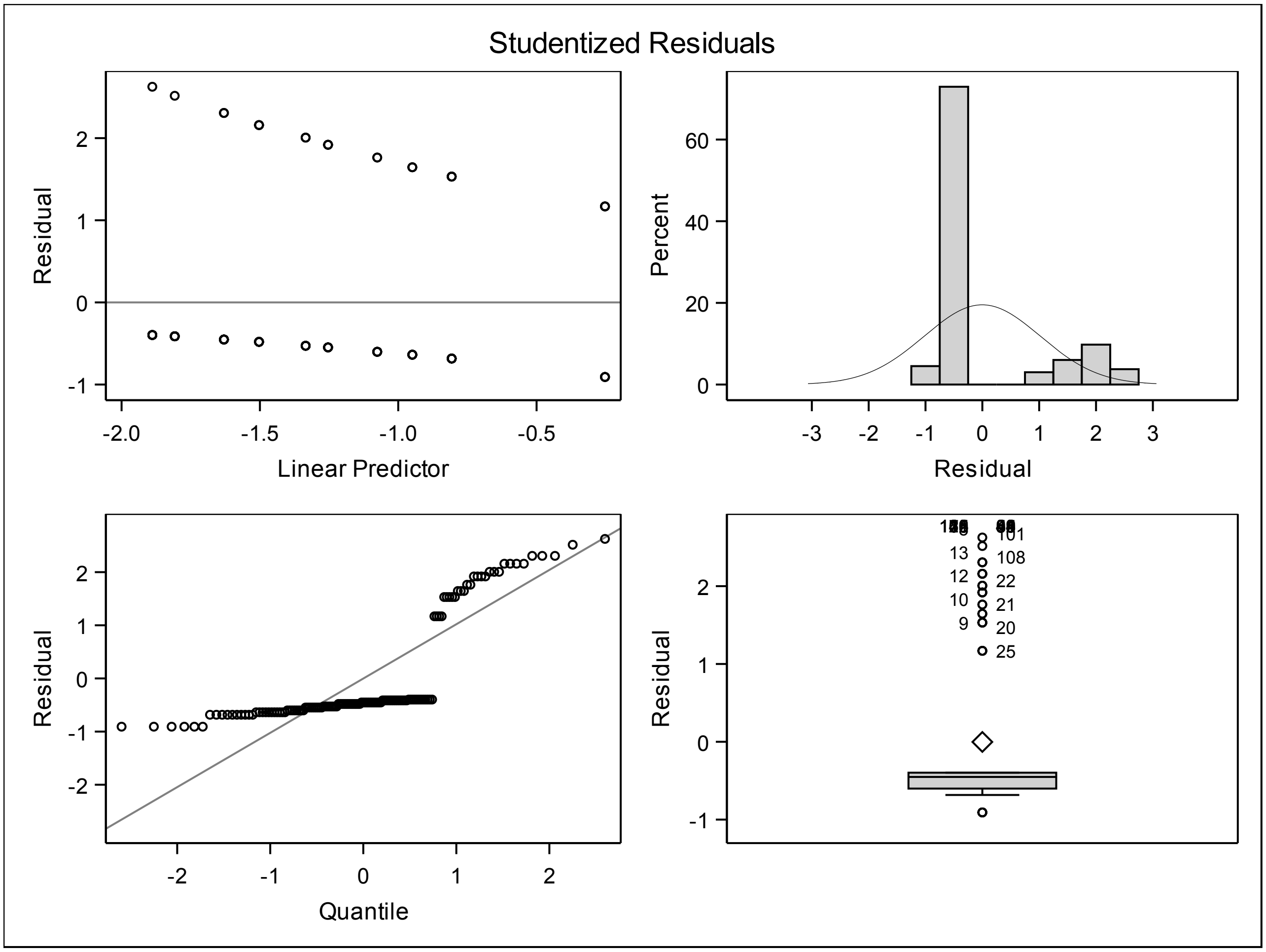

Mating and Fecundity- Length of successful parent galleries

The GLIMMIX Procedure

Model Information	
Data Set	WORK.MF
Response Variable	Total_gallery_length_cm
Response Distribution	Gaussian
Link Function	Identity
Variance Function	Default
Variance Matrix	Diagonal
Estimation Technique	Maximum Likelihood
Degrees of Freedom Method	Residual

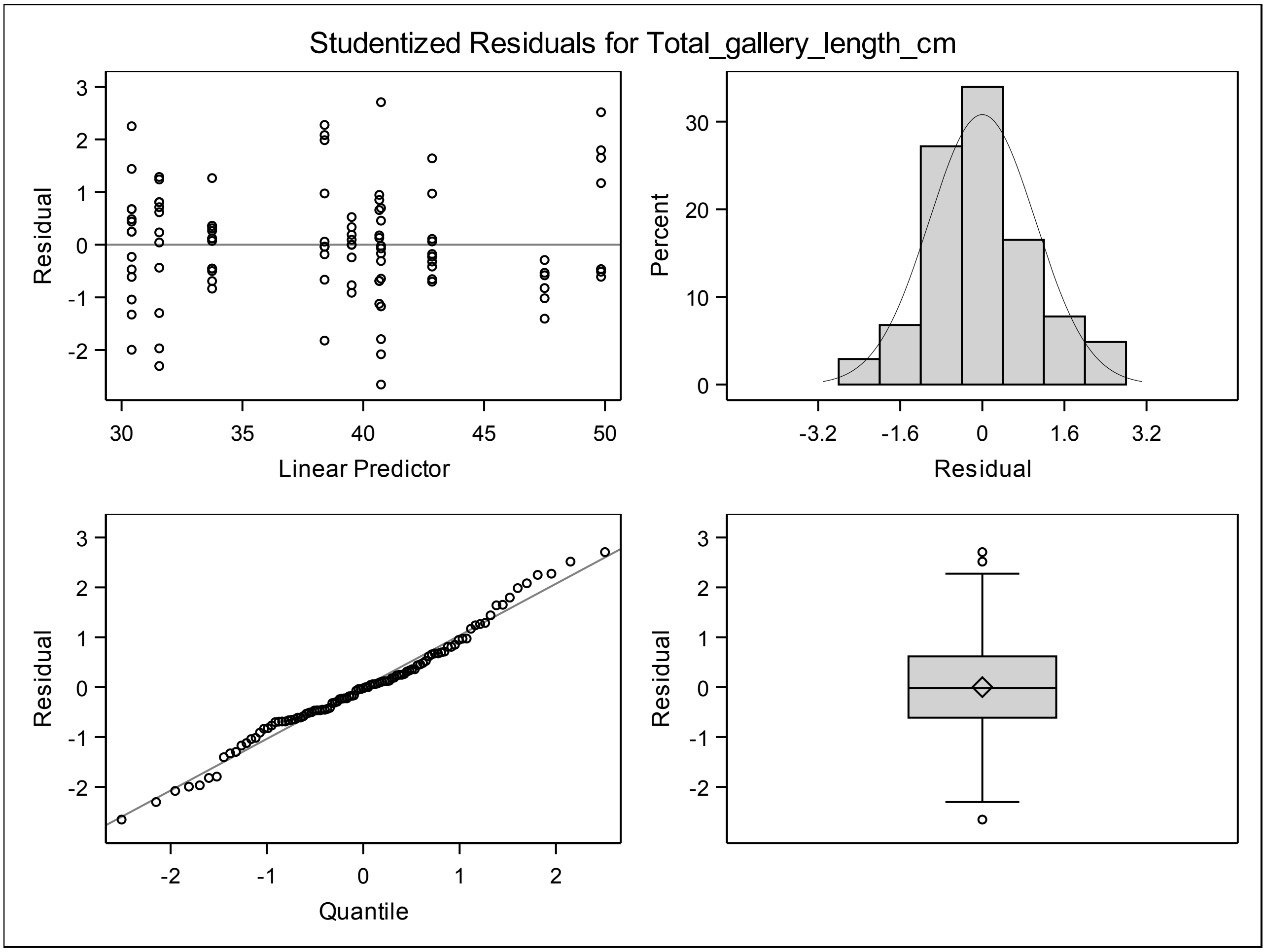

Mating and Fecundity- Total fecundity

The GLIMMIX Procedure

Model Information	
Data Set	WORK.MF
Response Variable	Sum_eggs_and_larvalgalleries
Response Distribution	Negative Binomial
Link Function	Log
Variance Function	Default
Variance Matrix	Diagonal
Estimation Technique	Maximum Likelihood
Degrees of Freedom Method	Residual

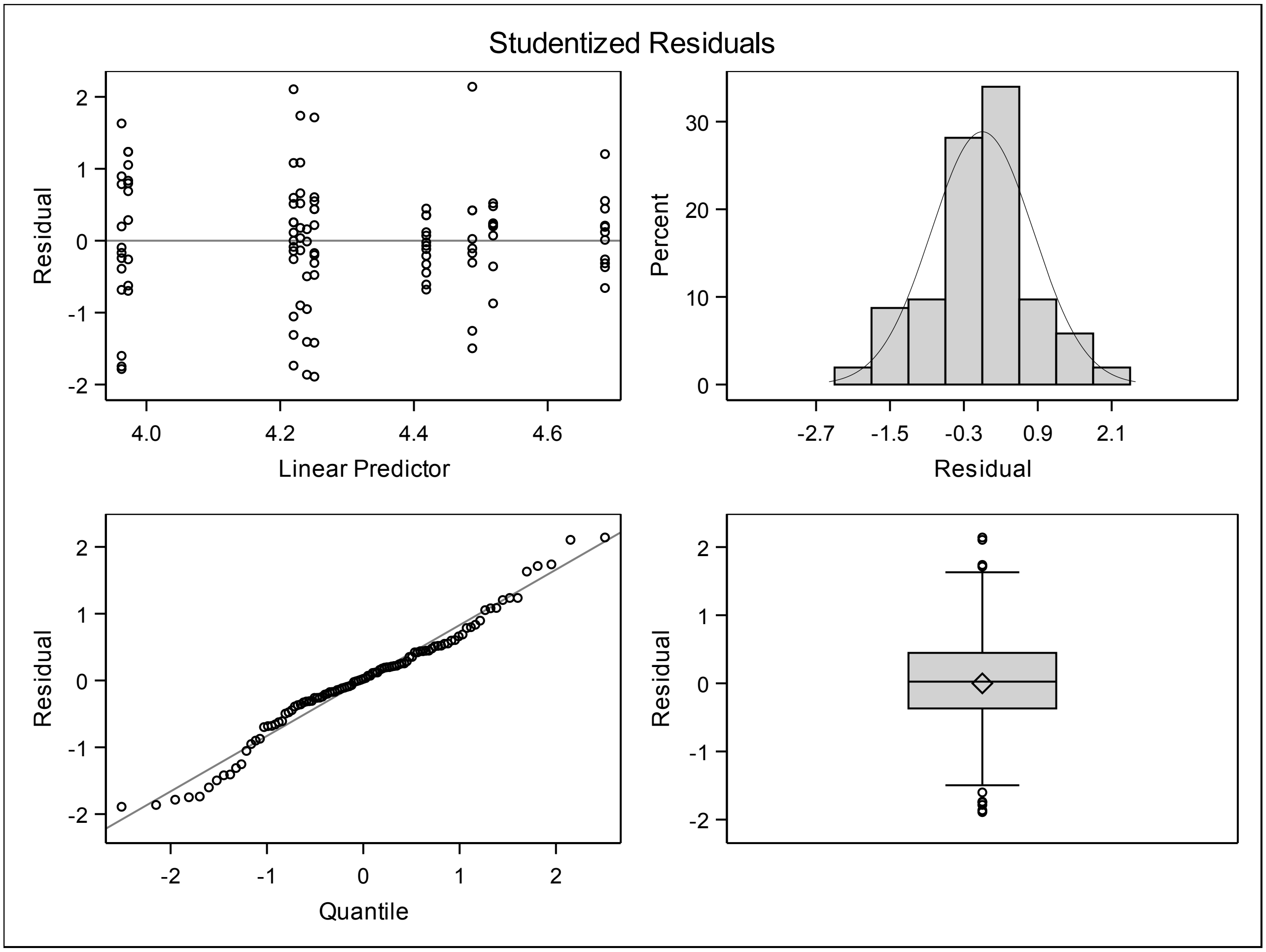

Offspring Emergence- Length of successful parent galleries

The GLIMMIX Procedure

Model Information	
Data Set	WORK.OGALS
Response Variable	Total_gallery_length_cm
Response Distribution	Gaussian
Link Function	Identity
Variance Function	Default
Variance Matrix	Not blocked
Estimation Technique	Maximum Likelihood
Likelihood Approximation	Laplace
Degrees of Freedom Method	Containment

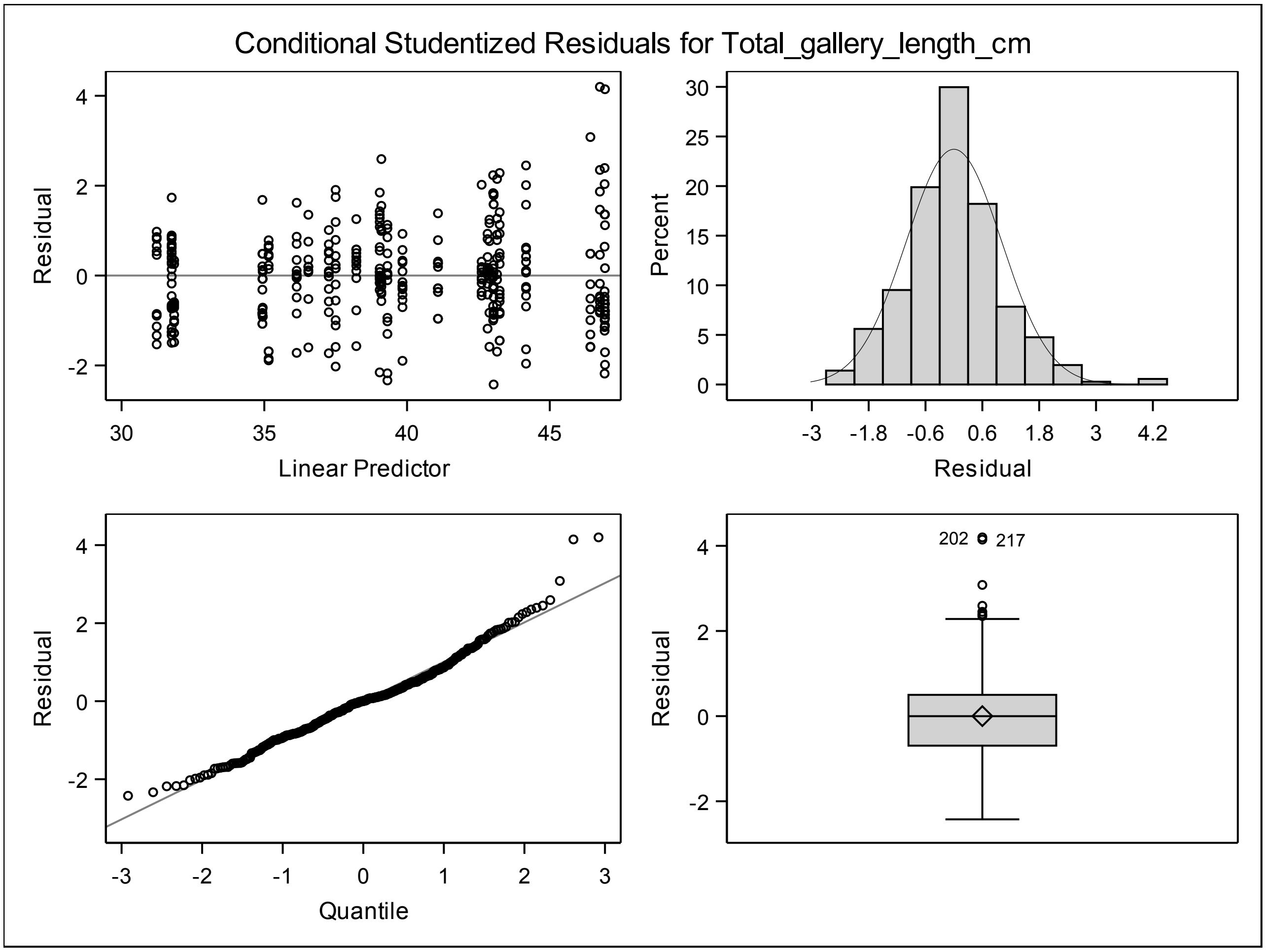

Offspring Emergence- Average total number of emerged offspring per successful parent gallery using offest

The GLIMMIX Procedure

Model Information	
Data Set	WORK.OEMERGE
Response Variable	Total_Offspring_Emerged
Response Distribution	Poisson
Link Function	Log
Variance Function	Default
Offset Variable	successful_galleries_log
Variance Matrix	Not blocked
Estimation Technique	Maximum Likelihood
Likelihood Approximation	Laplace
Degrees of Freedom Method	Containment

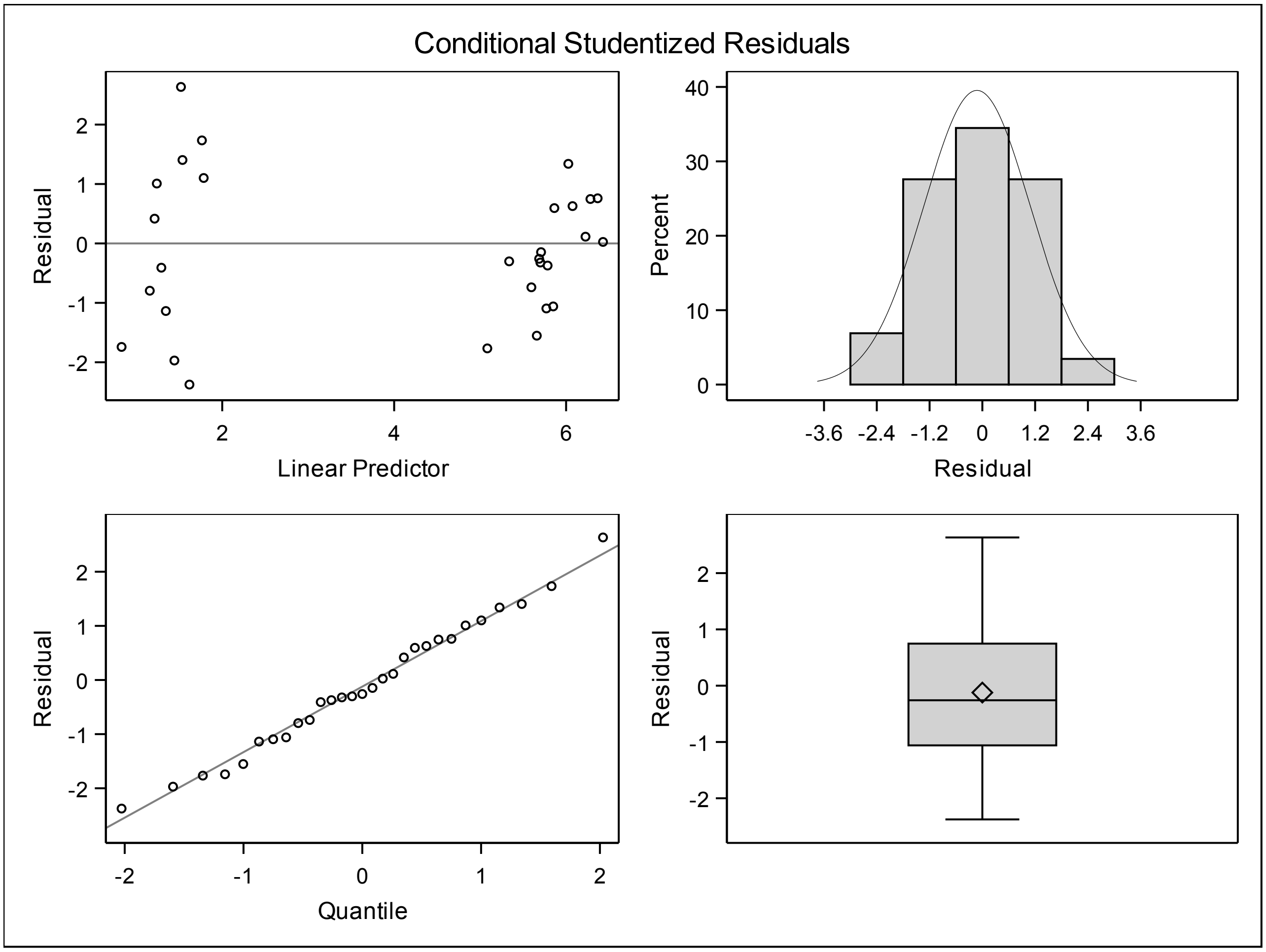

Offspring Emergence- Offspring size males

The GLIMMIX Procedure

Model Information	
Data Set	WORK.OSIZE
Response Variable	Offspring_Size
Response Distribution	Gaussian
Link Function	Identity
Variance Function	Default
Variance Matrix	Not blocked
Estimation Technique	Maximum Likelihood
Likelihood Approximation	Laplace
Degrees of Freedom Method	Containment

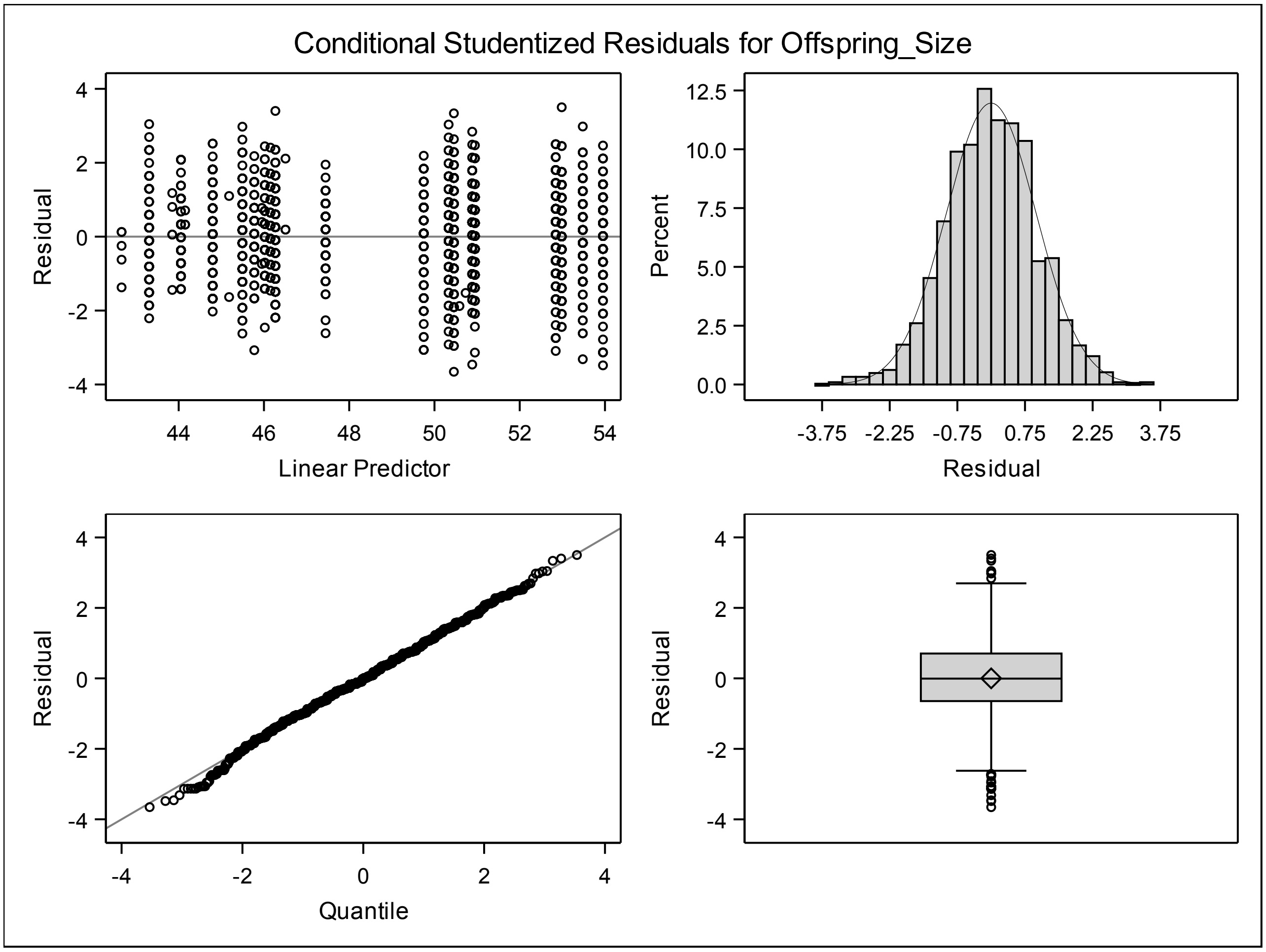

Offspring Emergence- Offspring size females

The GLIMMIX Procedure

Model Information	
Data Set	WORK.OSIZE
Response Variable	Offspring_Size
Response Distribution	Gaussian
Link Function	Identity
Variance Function	Default
Variance Matrix	Not blocked
Estimation Technique	Maximum Likelihood
Likelihood Approximation	Laplace
Degrees of Freedom Method	Containment

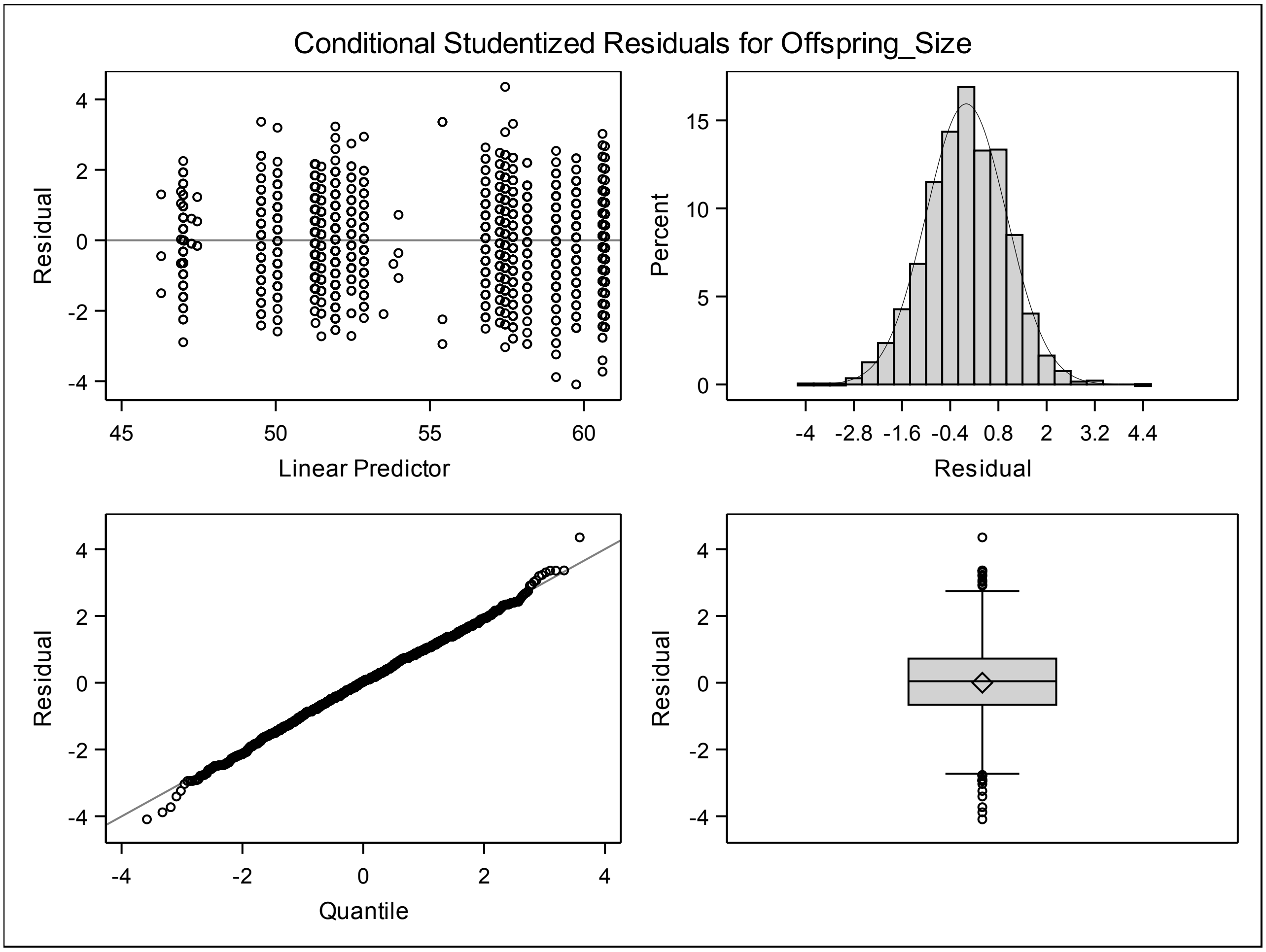

Supplement: S1 Supporting Information — Plots were generated using SAS Studio version 9.4. (PDF) [file pone.0196732.s003.pdf]
